# The immune landscape of SARS-CoV-2-associated Multisystem Inflammatory Syndrome in Children (MIS-C) from acute disease to recovery

**DOI:** 10.1016/j.isci.2021.103215

**Published:** 2021-10-02

**Authors:** Eleni Syrimi, Eanna Fennell, Alex Richter, Pavle Vrljicak, Richard Stark, Sascha Ott, Paul G. Murray, Eslam Al-Abadi, Ashish Chikermane, Pamela Dawson, Scott Hackett, Deepthi Jyothish, Hari Krishnan Kanthimathinathan, Sean Monaghan, Prasad Nagakumar, Barnaby R. Scholefield, Steven Welch, Naeem Khan, Sian Faustini, Kate Davies, Wioleta M. Zelek, Pamela Kearns, Graham S. Taylor

**Affiliations:** 1 Institute of Immunology and Immunotherapy, University of Birmingham, B15 2TT Birmingham, UK; 2 Health Research Institute and the Bernal Institute, University of Limerick, Limerick, Ireland; 3 Clinical Immunology Service, Institute of Immunology and Immunotherapy, University of Birmingham, B15 2TT Birmingham, UK; 4 Warwick Medical School, University of Warwick, Coventry, UK; 5 Bioinformatics Research Technology Platform, University of Warwick, Coventry, UK; 6 Birmingham Women's and Children's NHS Foundation Trust, Birmingham, UK; 7 Heartlands Hospital, University Hospitals Birmingham NHS Foundation Trust, Birmingham, UK; 8 Institute of Inflammation and Ageing, University of Birmingham, Birmingham, UK; 9 Systems Immunity Research Institute, School of Medicine, Cardiff University, Cardiff, UK; 10 NIHR Birmingham Biomedical Research Centre and Institute of Cancer and Genomic Sciences, University of Birmingham, Birmingham, UK

**Keywords:** Genomics, Immune response, Immune system disorder, Immunology

## Abstract

Multisystem inflammatory syndrome in children (MIS-C) is a life-threatening disease occurring several weeks after severe acute respiratory syndrome coronavirus 2 (SARS-CoV-2) infection. Deep immune profiling showed acute MIS-C patients had highly activated neutrophils, classical monocytes and memory CD8+ T-cells, with increased frequencies of B-cell plasmablasts and double-negative B-cells. Post treatment samples from the same patients, taken during symptom resolution, identified recovery-associated immune features including increased monocyte CD163 levels, emergence of a new population of immature neutrophils and, in some patients, transiently increased plasma arginase. Plasma profiling identified multiple features shared by MIS-C, Kawasaki Disease and COVID-19 and that therapeutic inhibition of IL-6 may be preferable to IL-1 or TNF-α. We identified several potential mechanisms of action for IVIG, the most commonly used drug to treat MIS-C. Finally, we showed systemic complement activation with high plasma C5b-9 levels is common in MIS-C suggesting complement inhibitors could be used to treat the disease.

## Highlights

• Granulocyte frequency correlates with MIS-C disease severity at presentation
• Recovering patients have increased CD163 on monocytes and new atypical neutrophils
• Cytokine profile of acute MIS-C suggests inhibiting IL6 rather than IL1 or TNF
• Raised plasma C5b-9 identifies complement inhibitors as potential MIS-C therapy

## Graphical abstract

**Figure:**
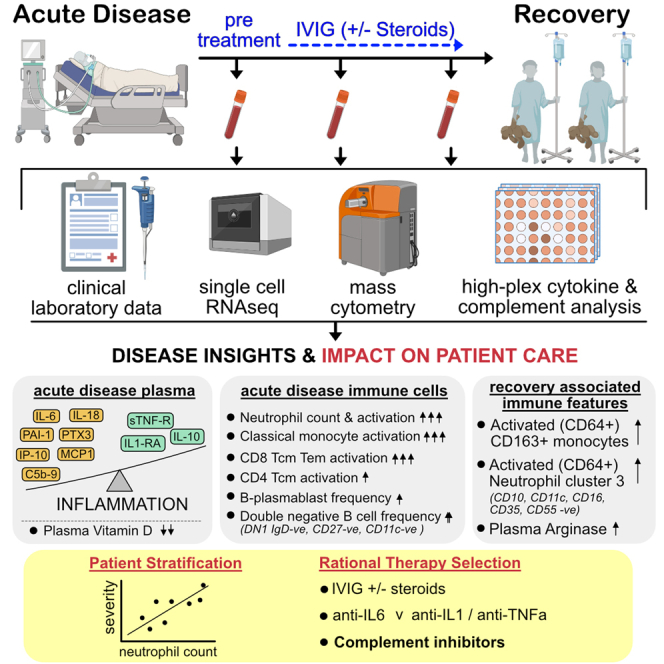

## Introduction

Infection of children with SARS-CoV-2, the viral cause of coronavirus disease 2019 (COVID-19) is associated with two distinct outcomes. The first is an acute infection of the respiratory tract that in most cases is asymptomatic or associated with mild respiratory symptoms (Castagnoli et al., 2020; Hoang et al., 2020). The second is a rare, severe hyperinflammatory syndrome called Multisystem Inflammatory Syndrome in Children (MIS-C) by the World Health Organization or pediatric inflammatory multisystem syndrome temporally associated with SARS-CoV-2 infection (PIMS-TS) in the UK (Ahmed et al., 2020; Kanthimathinathan and Scholefield, 2020; Whittaker et al., 2020; Hoste et al., 2021). Several weeks after the primary infection, children with MIS-C present with fever, inflammation and evidence of single or multi-organ failure that manifests with cardiac dysfunction, hypotension and life-threatening shock. This is accompanied by lymphopaenia and neutrophilia, both of which are rare in acute pediatric COVID-19 (Ahmed et al., 2020; Castagnoli et al., 2020; Hoang et al., 2020; Kanthimathinathan and Scholefield, 2020; Hoste et al., 2021).

MIS-C does, however, share clinical features with several pediatric inflammatory conditions including toxic shock syndrome (TSS), macrophage activation syndrome (MAS) and Kawasaki Disease (KD). KD is a systemic vasculitis that presents with symptoms of fever, rash, conjunctivitis, lymphadenopathy, and cardiac complications that are believed to be triggered by an as yet unidentified infectious agent (McCrindle et al., 2017; Rivas and Arditi, 2020). Acutely ill KD patients have increased blood levels of both proinflammatory anti anti-inflammatory cytokines with lymphopaenia and neutrophilia (McCrindle et al., 2017). Untreated KD can cause coronary aneurysms but the risk is substantially reduced by treatment with intravenous immunoglobulin (IVIG) (McCrindle et al., 2017). This agent is used to treat a diverse range of autoimmune and inflammatory conditions but its mechanism of action remains poorly defined (Nimmerjahn and Ravetch, 2008; Nagelkerke and Kuijpers, 2014). Although MIS-C shares features with KD there are also notable differences (Consiglio et al., 2020; Lee et al., 2020). For example, MIS-C patients often present with shock, cardiac dysfunction and hyperferritinemia, all of which are rarely seen in KD (McCrindle et al., 2017; Ahmed et al., 2020; Hoste et al., 2021).These differences suggest the underpinning pathology may differ between the two diseases potentially warranting different treatments.

The optimal treatment strategy for MIS-C is unknown and there are no widely accepted guidelines on patient management. IVIG is the most commonly used anti-inflammatory agent followed by systemic corticosteroids (Ahmed et al., 2020; Hoste et al., 2021) and targeted agents that selectively inhibit the interleukin (IL) −6, IL1-β or Tumor Necrosis Factor alpha (TNF-α) pathways in a smaller number of cases (Hoste et al., 2021; Ahmed et al., 2020; Lee et al., 2020; Consiglio et al., 2020; Carter et al., 2020; Rodríguez-Rubio et al., 2021; Diorio et al., 2020; Henderson and Yeung, 2021; Gruber et al., 2020). Identifying the abnormal immunological features present in acutely ill untreated MIS-C patients is therefore important not only to understand the pathogenesis of this new disease but also to inform rational treatment selection. We therefore performed high dimensional analysis of blood and plasma samples from children with MIS-C, comparing them to healthy children and SARS-CoV-2 negative KD patients recruited over the same time period. Analysis of longitudinal samples from patients identified multiple immunological changes occurring after treatment. Our results provide a detailed insight into the immunopathology of MIS-C from acute disease into recovery. They suggest that, amongst cytokine inhibitors, agents targeting IL-6 may be preferable to those that target IL1 or TNF-α. Furthermore, our data provide a rational basis for using complement inhibitors to treat MIS-C.

## Results

### Patient recruitment, treatment and clinical features

**Figure 1:**
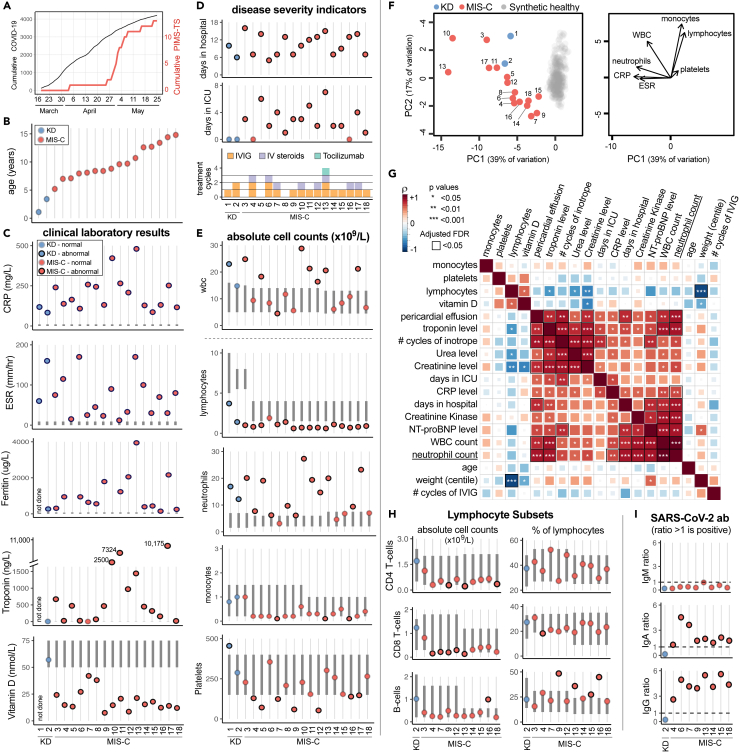
Demographic, clinical and immunological status of 18 pediatric patients with Kawasaki disease or MIS-C (A) Cumulative SARS-CoV-2 positive cases identified by PCR testing within the Birmingham area compared to MIS-C cases admitted to Birmingham Children's Hospital PICU. (B) Age of KD and MIS-C patients recruited to this study. (C) Clinical laboratory results shown for C-reactive protein (CRP), erythrocyte sedimentation rate (ESR), ferritin, troponin, and vitamin D. (D) Disease severity indicators shown as days hospitalized, days in PICU and treatment cycles of IVIG, intravenous steroids, and Tocilizumab. (E) Pretreatment absolute count of different immune cell subsets expressed as 10^9^ cells/L. (F) Left: Principal component analysis biplot of clinical laboratory features for patients with MIS-C or KD and synthetic healthy controls derived from normal range data. Right: Loading plot showing the top 7 features contributing to principal components one and two. (G) Correlation matrix of clinical features, immune parameters and demographics for the 16 MIS-C patients. The strength of each correlation is indicated by color and statistical significance by asterisks: ∗p < 0.05, ∗∗P < 0.01, ∗∗∗P < 0.001. Black outline indicates a significant result after 5% false discovery rate correction using the Benjamini-Hochberg method. (H) Pretreatment frequency of lymphocyte subsets expressed as the absolute number of cells x10^9^/L (left column) or percentage of total lymphocytes (right column). (I) SARS-CoV-2 ab responses for IgM, IgA, and IgG. In panels C, E, and H the normal range for each patient, based on their age, is shown by the vertical dark gray bar. Data points outside the normal range are drawn with a black outline. See also Figure S1.

Between late April and October 2020 at Birmingham Women and Children's Hospital, a tertiary level pediatric hospital, we recruited 16 children meeting the MIS-C diagnostic criteria established by the UK Royal College of Pediatrics and Child Health (Table S1). A surge of cases (50%) occurred approximately four weeks after SARS-CoV-2 cases were detected in the local community; subsequent cases accrued steadily over time (Figure 1A). All 16 MIS-C cases were over 5 years of age and tested positive for anti-SARS-CoV2 antibodies (Figure 1B and Table S1) (Perez-Toledo et al., 2020). Contemporaneously we recruited two patients with KD both of whom were under 5 years old and negative for anti-SARS-CoV-2 antibodies. Clinical laboratory tests showed all patients had elevated inflammatory markers, including ferritin (Figure 1C). Almost all MIS-C patients had high troponin and N-terminal pro B-type natriuretic peptides (NTpro-BNP, Figure 1C and data not shown). Interestingly, all MIS-C patients were deficient in vitamin D. Patients' length of hospital stay ranged between 5 and 16 days and 88% of MIS-C patients were admitted to the pediatric intensive care unit (PICU) from 2 to 8 days (Figure 1D). Of the 16 MIS-C patients, 13 received IVIG with three MIS-C patients (and one KD patient) receiving a second IVIG infusion because of ongoing inflammation. Eight MIS-C patients received IV methylprednisolone and one, patient 13, received anti-IL6 therapy (Tocilizumab). All 18 patients survived without long term cardiac complications at the time of last follow up.

### Clinical laboratory data shows neutrophilia is associated with severe disease in MIS-C

In accordance with previous reports (McCrindle et al., 2017; Ahmed et al., 2020; Feldstein et al., 2020; Hoste et al., 2021), both KD patients and almost all MIS-C patients had abnormally low lymphocyte and abnormally high neutrophils counts (Figure 1E). The absolute number of monocytes was normal for the KD patients but at the lower limit of normal for almost all MIS-C patients. Analysis of longitudinal data showed these perturbations resolved following treatment, although this was slower for granulocytes which were still elevated once lymphocytes and monocytes had returned to normal (Figure S1). Using principal component analysis to obtain a global view of the clinical laboratory data showed neutrophil count, CRP, and ESR correlated with each other with monocyte and lymphocyte counts orthogonal to these features (Figure 1F). All patients fell outside of the normal region to varying degrees.

We next investigated the relationships between clinical features, absolute immune cell counts and demographics for the MIS-C patients (Figure 1G) (Vella et al., 2021). Clinical markers of cardiac and kidney dysfunction (troponin, pericardial effusion, urea, and creatinine) positively correlated with use of vasoactive inotropes, consistent with these patients being in shock. We observed multiple highly significant positive correlations between absolute neutrophil count and markers of inflammation (CRP), cardiac dysfunction (presence of pericardial effusion, levels of troponin, creatine kinase, and NTpro-BNP) and overall length of hospital stay. Additional clinical laboratory assays performed on a subset of patients showed that, in accordance with lymphopenia, the absolute counts of CD4 and CD8 T-cells and B-cells were diminished. However, the relative proportion of these cells within the lymphocyte pool were generally unaltered (Figure 1H). Analyzing the anti-SARS-CoV-2 antibody response in detail for eight MIS-C patients showed all eight had IgG and IgA antibodies but lacked IgM antibodies consistent with MIS-C developing weeks after virus infection occurred (Figure 1I) (Castagnoli et al., 2020; Hoang et al., 2020; Hoste et al., 2021).

### scRNA sequencing data show monocytes are profoundly altered in MIS-C and KD patients

**Figure 2:**
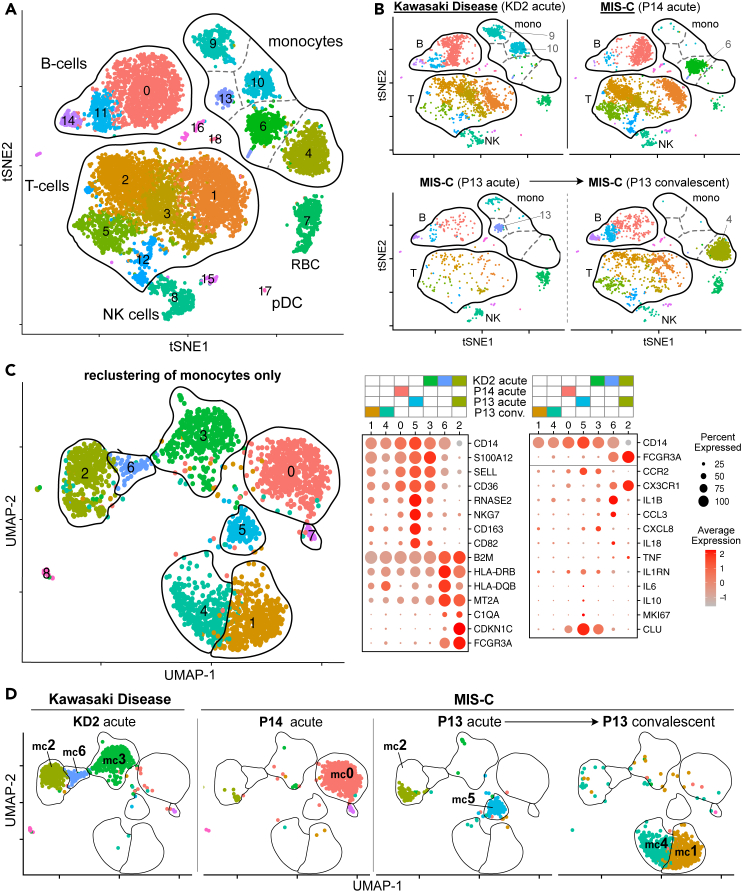
Single cell RNA sequence analysis of MIS-C and KD PBMC (A) tSNE representation of major cell types and associated FlowSOM clusters in acute stage PBMCs from patients KD2, P13, and P14 and a convalescent sample from P13 at discharge from PICU. All four samples were processed and sequenced in a single experiment. (B) tSNE representation of PBMCs from each patient sample. (C) UMAP representation of monocyte cells after re-clustering on monocytes alone in acute stage samples from patients KD2, P13, and P14 and a convalescent sample from P13 at discharge from PICU. Right hand panel shows the expression level of selected genes within each cluster. (D) UMAP representation of monocyte clusters from each patient sample. See also Figures S2–S8.

To explore immune changes in MIS-C in more detail, we first performed an unbiased analysis by performing single cell RNA sequencing (scRNAseq) on acute stage peripheral blood mononuclear cells (PMBCs) of two representative patients (P13 and P14) with MIS-C. Both patients were admitted to PICU, but patient P14 responded rapidly to one cycle of IVIG and stayed on PICU for three days whereas patient P13 stayed on PICU for eight days and received a second IVIG infusion, intravenous steroids, and Tocilizumab, a monoclonal antibody against the interleukin-6 receptor, to control their disease. The acute stage samples were collected before immune modulating treatment commenced (P13) or eight hours after IVIG infusion (P14). A convalescent sample from P13 collected at discharge from PICU was also analyzed. For comparative purposes we also analyzed pre-treatment acute-stage PBMCs from patient KD2 who required two infusions of IVIG (Figure 1D). All four samples were thawed, processed and sequenced in the same experiment. Unsupervised clustering of 10,031 cells produced 19 different clusters comprising all major lymphocyte subsets (Figures 2A and S2–S4). Analyzing each patient separately, we observed that all possessed lymphocytes assigned to one of the nine B-cell, T cell, or NK cell clusters although the frequencies of cells within each of these clusters varied between patients and, for P13, from acute disease to convalescence (Figure 2B). In contrast, each patient's monocytes were assigned to only one or two of the five different monocyte clusters. Four of these clusters corresponded to CD14^+^ classical monocytes and for each sample these cells were assigned to a separate cluster Based on CD14 and CD16 expression the fifth monocyte cluster (cluster nine) contained intermediate and non-classical monocytes, and these varied markedly in frequency between samples: abundant in the acute sample from patient KD2, less frequent in the acute sample from patient P13 and scarce in the convalescent sample from this same patient but also the acute sample from P14. The marked changes we observed in monocyte populations between patients, with other cell types comparatively inert, suggested monocyte-specific changes were present rather than a batch effect.

Pathway analysis (Figure S5) on genes upregulated in each of the major immune subsets, relative to the same subset in the convalescent sample from P13, showed highly significant enrichment of genes annotated with GO-term GO:0002446 ‘Neutrophil mediated immunity’ in CD14^+^ monocytes from patient KD2 (p = 3x10^−34^) and P13 (p = 7x10^−31^). Although labeled as neutrophil mediated immunity, this pathway contains many genes expressed by monocytes. Genes upregulated in CD14^+^ monocytes from KD2 and P13 included: genes involved in complement function and regulation (*CD35* and *CD55*); adhesion, homing and scavenger receptors (*CD36*, *CD62L*, and *CD63*); Fc receptors (*FCGR2A* and *FCER1G*); alarmin related S100A molecules (S*100A8*, *S100A9*, *S100A11,* and *S100A12*) and regulation of innate cell mediated inflammation (*SERPINB1*). Because of the lack of intermediate/non-classical monocytes in the convalescent sample we did not perform GO-term analysis for this subset.

Repeating dimensionality reduction and clustering analyses on just the subset of data from the four samples that represented monocytes yielded nine monocyte clusters (numbered mc0 to mc8) that could now be assigned to the three canonical monocyte subsets (Wong et al., 2011) (Figures 2C and S6). Clusters mc0, mc1, mc3, mc4, and mc5 were CD14^+^ CD16^-^ classical monocytes (CM), mc6 (CD14^int^ CD16^int^) comprised intermediate monocytes (IM) while mc2 (CD14^lo^ CD16^hi^) comprised non-classical monocytes (NCM). Previous studies have shown that the large majority of monocytes in the blood of healthy children are CM with only small populations of IM and NCM present (Wong et al., 2011).This distribution was distorted in the acute KD sample with high frequencies of IM (cluster mc6, 8%) and NCM (cluster mc2, 38%) (Figure 2D). The acute sample from MIS-C patient P13 also had an abnormally high frequency of NCM (34% of total monocytes) but lacked IM. Upon recovery, monocytes from MIS-C patient P13 returned to the normal state with only the CM population present in their convalescent sample. Examining gene expression in more detail (Figures 2C and S7) we detected mRNA encoding IL-1β in the mc6 intermediate monocytes abundant in patient KD2 but detected low or no transcripts encoding other monocyte-associated cytokines including IL-6, IL-8 (gene *CXCL8*), IL-10, IL-18, TNF-α or IL1 receptor antagonist (IL-1RA, gene *IL1RN*).

### Mass cytometry shows monocytes granulocytes and CD8 memory T-cells are contemporaneously activated in MIS-C and KD patients

To examine the above changes in more patients and to extend our analysis to granulocytes, which we and others have shown are abnormally expanded in MIS-C and KD patients' blood (Ahmed et al., 2020; Hoste et al., 2021), we used the scRNAseq analysis as a guide to develop a MIS-C focused 38 marker mass cytometry panel (Table S2) and used this to investigate whole blood samples from 7 patients (6 MIS-C and 1 KD) and 7 healthy children. The former included patients P13, P14 and KD2 (whose PBMCs were examined by scRNA sequencing) and four additional MIS-C patients from our cohort. Samples were collected during the acute stage, two days after IVIG administration and upon discharge from PICU or hospital (Figure S8). Acute stage samples were taken before IVIG with one exception, the sample from P14, which was collected 8 hours after IVIG infusion.

**Figure 3:**
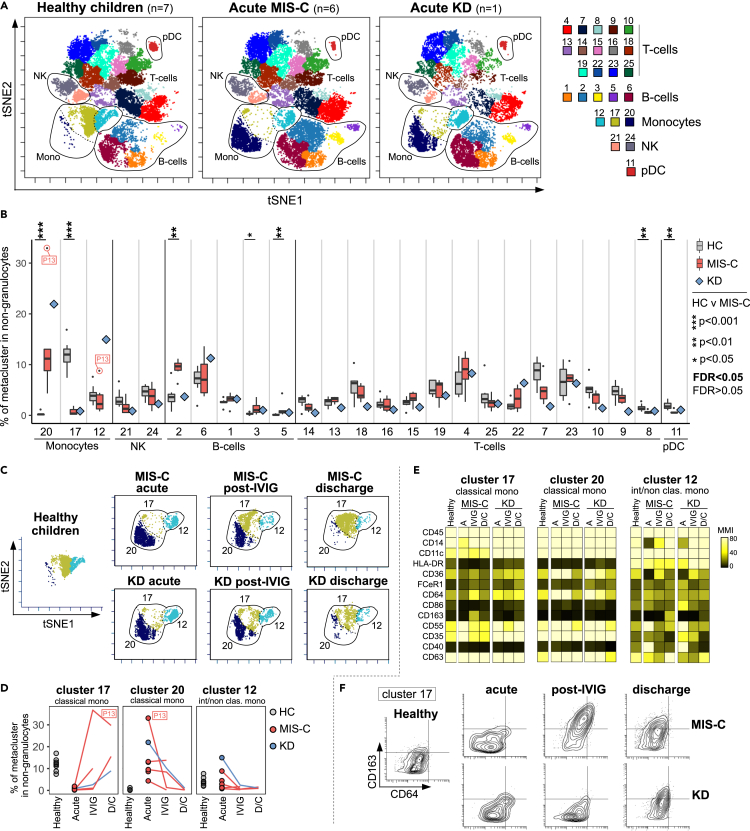
Mass cytometry analysis of mononuclear cells in whole blood samples from healthy children and patients with MIS-C or KD (A) tSNE plots of concatenated flow cytometry data from six MIS-C patients or one KD patient at the acute stage of their disease alongside seven healthy children (HC). Each meta-cluster is represented by a different color and key populations are indicated on the plots. Results from these concatenated datafiles are shown throughout this figure. (B) The frequency of each FlowSOM metacluster in the same donors expressed as a percentage of total non-granulocyte mononuclear cells are shown as box and whisker plots (healthy children and MIS-C) or a blue diamond (KD patient KD2). Results of Wilcoxon rank-sum tests comparing the frequency of each cluster in healthy children to the frequency in acute MIS-C patients are indicated by: ∗p < 0.05, ∗∗p < 0.01, ∗∗∗p < 0.001. Non-significant results are not shown and emboldened p value symbols indicate significant results after 5% false discovery rate correction using the Benjamini-Hochberg method. (C) tSNE plots showing cells within monocyte clusters 17, 20 and 12 for MIS-C patients at the acute stage, post IVIG and at discharge from PICU alongside plots showing cells from a single KD patient or seven healthy children. (D) Trajectory of each of the three monocyte clusters over time in seven healthy children or MIS-C patients over time (acute stage, post IVIG, and PICU discharge). Data from patient P13 is indicated on the plots. (E) Heatmaps showing the median metal intensity (MMI) of markers expressed on monocyte clusters 17, 20, and 12. (F) Biaxial plots of CD64 and CD163 expression on cluster 17 monocytes cells in healthy children or patients with MIS-C or KD at the acute, post-IVIG, or PICU/hospital discharge stages of disease. Note that all data in the Figure were generated from the same seven healthy children, six MIS-C patients (6 acute stage, 4 post IVIG, and 2 discharge samples) or a single KD patient. See also Figures S8–S10.

Unsupervised dimensionality reduction and clustering of 224,000 cells (16,000 from each of the seven healthy children, six MIS-C patients and one KD patient) identified 24 clusters comprising plasmacytoid dendritic cells (pDCs), T-cells, B-cells, NK-cells, and monocytes (Figures 3A and S9). Comparing the combined data from 7 healthy children to 6 acutely ill patients we observed profound changes in monocyte cluster abundance as noted in the scRNAseq data analysis. We next evaluated the data for each individual (Figure 3B). The frequency of seven clusters was significantly different in MIS-C patients compared to healthy children. Note that we chose not to include the KD patient in this statistical analysis but show their results on the plot as an exemplar of this disease. Based on marker expression (Figure S9) the frequency of activated CM (cluster 20) was significantly increased in MIS-C with a concomitant decrease of non-activated CM (cluster 17). There was no significant difference in the frequency of both IM and NCM (cluster 12), but we noted MIS-C patient P13 and KD patient KD2 had high frequencies of these cells consistent with their scRNAseq data. Examining other immune cell types, MIS-C patients showed a small but significant increase in CD19^+^ CD38^hi^ CD27^hi^ B-cell plasmablasts (cluster 5) and a much larger increase in IgD-CD27-double-negative (DN) B-cells (cluster 2). These DN B-cells lacked CD11c consistent with them being the recently proposed DN1 B-cell subset (Sanz et al., 2019). T cell cluster 8 and pDCs (cluster 11) were both decreased in MIS-C patients relative to controls.

We next examined the immune features associated with disease recovery. IVIG acts rapidly, resolving inflammation in most KD patients two days after infusion although a minority requires additional treatments because of ongoing inflammation or recrudescence (Newburger et al., 1991). We therefore re-sampled our patients two days after IVIG infusion and then upon discharge from ICU or hospital (Figure S8). Compared to their pre-treatment samples, monocytes in the post-IVIG samples had started to normalize with decreases in the frequency of cells in activated CM and IM/NCM (cluster 20 and 12, respectively) and increases in the frequency of non-activated CM (cluster 17). This reversion to normality continued further to PICU discharge, at which point the patients' classical monocyte cluster distributions resembled those of healthy children (Figures 3C and 3D). Reversion proceeded rapidly for patient P13, who had the highest frequency of activated CM (cluster 20) at the acute stage. Examining the phenotype of each monocyte cluster over time, we observed that levels of CD163 increased on the non-activated CM cells (cluster 17) after IVIG (Figure 3E). Interestingly the level of CD64 also increased on these cells at this time (albeit to a lower degree than the highly activated cluster 20 CM cells) suggesting the CD163-positive CM had also undergone a degree of activation. The CD163 CD64 double-positive cells were present in 3 of the 4 MIS-C patients who received IVIG and from whom we obtained post-IVIG samples (Figure S10). They were also present in the discharge sample from KD patient KD2 which was collected two days after a second cycle of IVIG was administered because of ongoing inflammation. Thus, their appearance was coincident with ongoing disease resolution (Supplemental information Figure S8).

**Figure 4:**
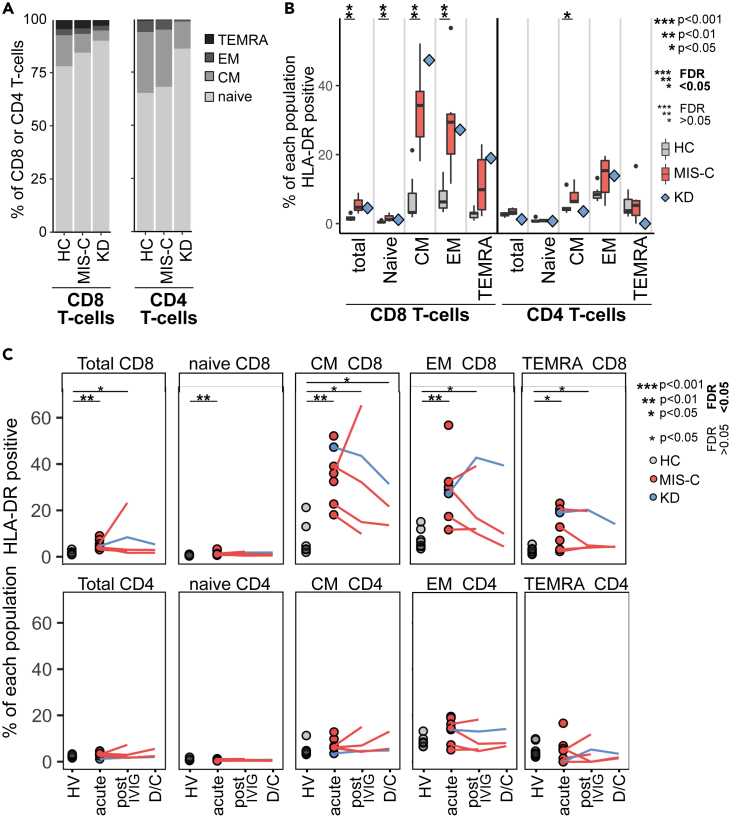
Mass cytometry of mononuclear cells analyzed by manual gating on canonical T cell subpopulations (A) Percentage of CD8+ and CD4+ T-cells in each of the four canonical T cell sub-populations for seven healthy children, six acute MIS-C patients or a single acute KD patient. (B) The percentage of each T cell subpopulation (from the same donors shown in panel A) that were positive for HLA-DR are shown as box and whisker plots (health donors or MIS-C patients) or a blue diamond (KD patient KD2). (C) Percentage of each T cell subpopulation positive for HLA-DR over the course of disease. For all panels the data were from seven healthy children, six MIS-C patients (6 acute stage, 4 post IVIG and 2 discharge (D/C) samples) or a single KD patient. In panels B and C the results of Wilcoxon ranked sum tests comparing the frequency of each cluster in healthy children to MIS-C patients (acute stage only in panel B or acute, post-IVIG or discharge stages in panel C) are indicated: ∗p < 0.05, ∗∗p < 0.01, ∗∗∗p < 0.001. Non-significant results are not shown. Emboldened p value symbols indicate significant results after 5% false discovery rate correction using the Benjamini-Hochberg method. See also Figure S11.

Examining the expression of different proteins across the fourteen T cell clusters (Figure S9) we noted most T-cells in MIS-C patients were CD45RA^+^ CD27^+^ naive cells. One cluster of CD8 non-naïve T-cells (cluster 13, with low CD45RA and CD27 expression) expressed HLA-DR, a marker of T cell activation(Maecker et al., 2012).To explore this in more detail we used CD27 and CD45RA to manually gate CD8^+^ and CD4^+^ T-cells into the four canonical T cell subgroups: naive (Tn), central memory (Tcm), effector memory (Tem) and terminally differentiated effector memory re-expressing CD45RA (TemRA) (Sallusto et al., 1999) (Figure S11). As expected for children, and consistent with the unsupervised clustering, we found all healthy donors and patients had a high proportion of naive T-cells (Figure 4A) with the highest in the KD patient likely due to their younger age (Taylor et al., 2019; Lakshmikanth et al., 2020). Comparing healthy children to MIS-C patients we found no significant differences in the distribution of the four subgroups in CD8^+^ or CD4^+^ T-cells (Figure S11C). However, MIS-C patients had a significantly higher proportion of HLA-DR positive activated CD8-T-cells at the acute stage of disease (Figure 4B). In MIS-C patients, only 2% of Tn cells were HLA-DR positive whereas 35% of Tcm and 30% of Tem CD8^+^ T-cells were HLA-DR positive. The proportion of HLA-DR positive CD8^+^ T-cells remained high after IVIG and, for one patient (P7), increased markedly (Figure 4C). At discharge, the proportion of activated cells had declined but was still higher than controls. In contrast, only a small proportion of CD4^+^ T-cells expressed HLA-DR and only Tcm were significantly higher than controls at the acute stage. The KD patient showed the same pattern of HLA-DR expression on their CD8^+^ and CD4^+^ T cell subsets.

**Figure 5:**
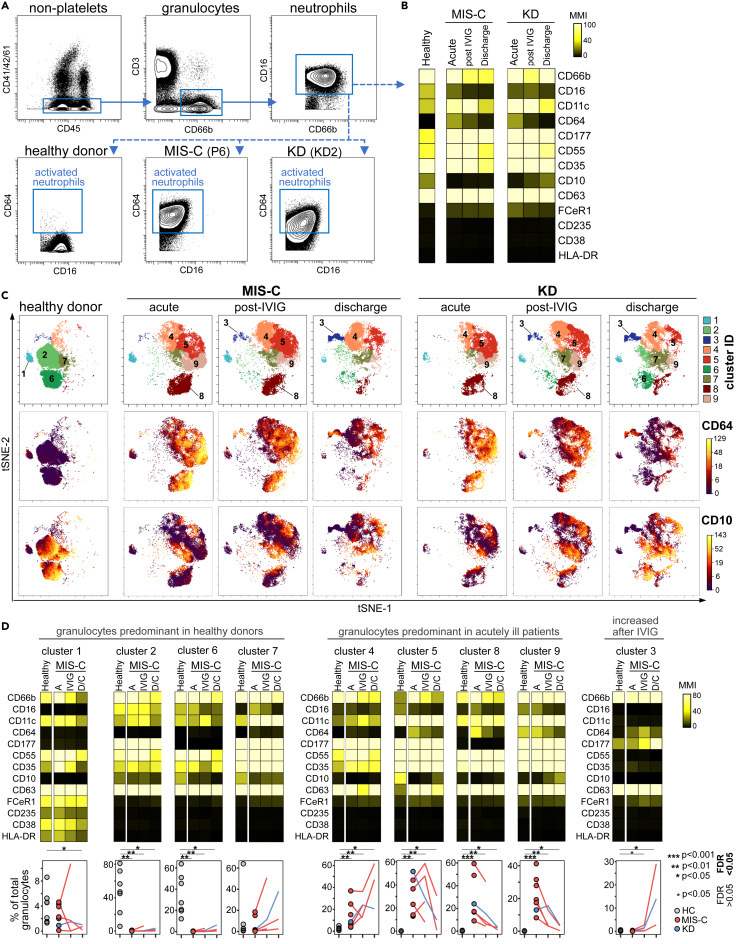
Mass cytometry analysis of granulocytes in whole blood samples from healthy children and patients with MIS-C or KD (A) Gating strategy used to manually gate and analyze neutrophil activation in whole blood. (B) Heatmaps showing median metal intensity (MMI) of markers expressed on manually gated neutrophils. Data are from concatenated FCS files from seven healthy children, six MIS-C patients (acute n = 6, post-IVIG n = 4, discharge n = 2) and a single KD patient (KD2). (C) tSNE plots of granulocytes from the same donors analyzed by unsupervised clustering. Top row: FlowSom metaclusters. Middle row: CD64 expression. Bottom row: CD10 expression. (D) Upper panels: heatmaps showing expression level of different markers in each metacluster for the same donors. Lower panels: Trajectory of each metacluster over time, expressed as a percentage of total granulocytes, for each of the healthy children and patients. The results of Wilcoxon ranked sum tests comparing the frequency of each cluster in healthy children to seven patients (six MIS-C and one KD patient) at the acute (n = 7), post-IVIG (n = 5) and discharge (n = 3) timepoints as indicated: ∗p < 0.05, ∗∗p < 0.01, ∗∗∗p < 0.001. Non-significant results are not shown and emboldened p value symbols indicate significant results after 5% false discovery rate correction using the Benjamini-Hochberg method. See also Figure S8.

Turning to granulocytes, we manually gated and examined the CD66b^+^ CD16^+^ neutrophil population (Figure 5A). We compared equal numbers of cells (22,000) sampled from seven healthy children or from MIS-C patients at each of three key timepoints (6 acutely ill, 4 after IVIG and 2 at time of discharge); for comparison we also examined cells from these same timepoints from patient KD2 (Figure 5B). Both patient groups exhibited the same changes in phenotype, with decreased expression of the granulocyte maturity markers CD16 and CD10 (Marini et al., 2017)^,^ and increased expression of the neutrophil activation marker CD64 (Nagelkerke and Kuijpers, 2014), which was highest at the acute stage then slowly decreased after IVIG and at discharge, although levels were still raised at this time.

Unsupervised dimensionality reduction and clustering of the total granulocyte population identified nine clusters (Figure 5C). One cluster, cluster 1, present in patients and healthy children were eosinophils based on expression of FceR1, CD38, HLA-DR and lack of CD16 and CD10 (Figure 5D). The other 8 clusters were neutrophils and these showed marked differences in abundance. Healthy children had few cells classified into clusters 4,5,8 and 9, whereas almost all cells in acutely ill MIS-C patients belonged to these clusters. This redistribution of neutrophils was driven by a dramatic decrease in CD10 and increase in CD64 on patients' neutrophils. Expression of both markers had begun to return towards healthy children's levels after IVIG administration then had further normalized at discharge, although the frequencies of all four activated clusters (clusters 4, 5, 8, 9) were still significantly higher at this time. In contrast, eosinophils (cluster 1) showed only modest changes in frequency and phenotype with only CD35 (complement receptor 1) expression varying over time.

Granulocyte cluster 3 was present at very low frequency in healthy children (median frequency 0.16% of granulocytes), and in acutely ill MIS-C (0.11%) and KD (0.04%) patients. However, after IVIG the frequency of these cells increased 5 to 12-fold over pretreatment values in the MIS-C patients (median frequency 0.81% range 0.30–2.7%) and 30-fold (frequency 1.6%) in the KD patient. These cells continued to increase in frequency over time and at discharge their frequency was 70-fold–204-fold higher than at the acute stage. At discharge they comprised 2.79% and 29.05% of total granulocytes in MIS-C patients P6 and P13 respectively. This continued increase also occurred in the KD patient (KD2) with cluster 3 cells having a frequency 280-fold higher in the discharge sample compared to the acute sample, comprising 14.0% of this patient's granulocytes at discharge. Cluster 3 cells also possessed an unusual phenotype; they were clearly granulocytes based on their strong expression of the canonical granulocyte marker CD66b with their lack of CD16 and CD10 indicating immaturity. However, they were different to all other granulocyte clusters as they lacked expression of CD11c, CD35, and CD55. Cluster 3 granulocytes in MIS-C patients and the KD patient, but not healthy children, expressed CD64 indicating these cells were not only increased in frequency but were also activated during the course of disease.

### Proinflammatory and anti-inflammatory cytokines are elevated in MIS-C and KD patients and arginase is increased after IVIG administration

**Figure 6:**
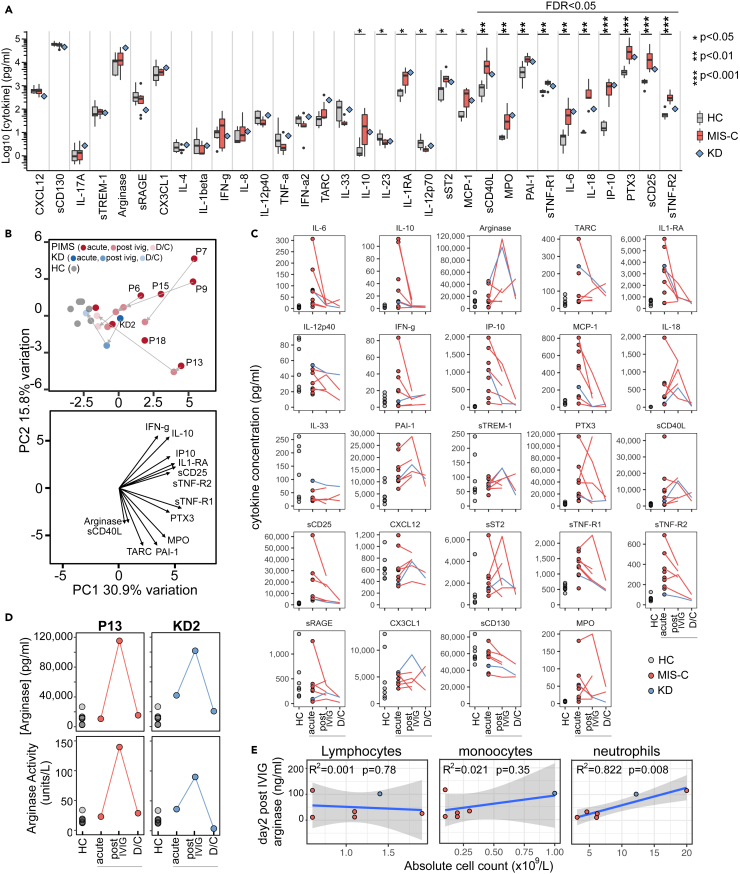
Analysis of cytokines in plasma samples from healthy children and patients (A) Levels of cytokines in plasma from seven healthy children or eight MIS-C patients at the acute stage of disease are shown as boxes and whiskers alongside a blue diamond indicating results from a single KD patient (KD2) also at the acute stage. Results of Wilcoxon rank-sum tests comparing the concentration of each cytokine in seven healthy children to the concentration in eight acute MIS-C patients are indicated by: ∗p < 0.05, ∗∗p < 0.01, ∗∗∗p < 0.001. Non-significant results are not shown and emboldened p value symbols indicate significant results after 5% false discovery rate correction using the Benjamini-Hochberg method. (B) Upper panel: principal component analysis biplot of cytokines. Lower panel: loading plot showing the top 13 features contributing to principal components one and two. (C) Trajectory of cytokines over time for the same healthy children and patients shown in panel A at the acute, post-IVIG and discharge timepoints. No statistical testing was performed. (D) Plots showing the concentration (upper panel) and enzyme activity (lower panel) of arginase over time in plasma samples from seven healthy children, MIS-C patient P13 and KD patient KD2. (E) Results of linear regression analysis of the acute disease stage absolute counts of lymphocytes, monocytes or neutrophils against the plasma arginase concentration after IVIG treatment. The R^2^ and statistical significance of each regression model is shown on the plot with the shaded area indicating the 95% confidence interval. See also Figure S8.

We next performed a wide-ranging analysis of 32 cytokines and chemokines in plasma samples from nine patients (eight MIS-C, one KD) and seven healthy children. These soluble immune mediators were selected based on the cellular changes we observed in MIS-C and KD as well as prior studies on KD and, more recently, MIS-C patients(Lee et al., 2020; Consiglio et al., 2020; Carter et al., 2020; Rodríguez-Rubio et al., 2021; Diorio et al., 2020; Ko et al., 2015; Ching et al., 2020; Ren et al., 2015; Takeshita et al., 1999; Weng et al., 2013; Hokibara et al., 2016; Gruber et al., 2020). We selected assays capable of providing absolute quantification to allow data from our patients to be directly compared with historical data from KD and other inflammatory conditions. Comparing MIS-C patients to healthy children, we identified statistically significant differences for 16 of the 32 soluble mediators analyzed (Figure 6A). MIS-C patients had significantly increased levels of the chemokines monocyte chemoattractant protein 1 (MCP-1/CCL2) and interferon gamma-induced protein 10 (IP-10/CXCL10), higher levels of the pro-inflammatory cytokines IL-6 and IL-18, but at the same time, higher levels of anti-inflammatory cytokine IL-10. Soluble receptors of tumor necrosis factor alpha (sTNF-R1 and sTNF-R2), CD40 ligand (sCD40L) and IL-2 (sCD25) were all higher in MIS-C patients as was interleukin-1 receptor antagonist (IL-1RA), a member of the IL1 family that binds the IL1-receptor to inhibit this pathway. Plasminogen activator inhibitor 1 (PAI-1), pentraxin-3 (PTX3) and myeloperoxidase (MPO) were also higher in MIS-C patients. For several pro-inflammatory cytokines and chemokines there was no difference between patients or the healthy donor controls, including: IL1-β, IL-8 (CXCL8), IL-17A, interferon-alpha2 (IFN-a2) interferon-gamma (IFN-γ), and TNF-α. We did not include the KD patient in the statistical analysis, but their acute blood sample had the same profile of cytokines, chemokines and other soluble factors as the acute MIS-C patients (Figure 6A).

Principal component analysis divided patients into two broad groups (Figure 6B). Acute MIS-C samples were most distant from the healthy children and almost all patients migrated toward the healthy state after IVIG therapy, the exception being P13, a patient with particularly severe disease. Examining each soluble mediator over the disease course (Figure 6C) we found that many decreased following treatments with IVIG or IVIG combined with steroids (Figure S8). These included both proinflammatory (IL-6, IP-10, MCP-1) and anti-inflammatory (IL-10, IL-1RA) molecules. A notable exception was arginase, levels of which in the acute phase of disease were not significantly higher than those in healthy children but increased dramatically for MIS-C patient P13 and patient KD2 in their post IVIG sample. Patient P13 received IVIG and then steroids before this sample but patient KD2 received IVIG alone. The increased quantity of arginase in these patients' plasma was confirmed to be enzymatically active in an independent assay (Figure 6D). Finally, across all patients we noted a significant positive correlation between post-IVIG arginase levels and pre-treatment absolute number of neutrophils (r = 0.91, R^2^ = 0.822, p = 0.008), the main source of arginase in humans (Munder et al., 2005) but no correlation with lymphocytes or monocytes (Figure 6E).

**Figure 7:**
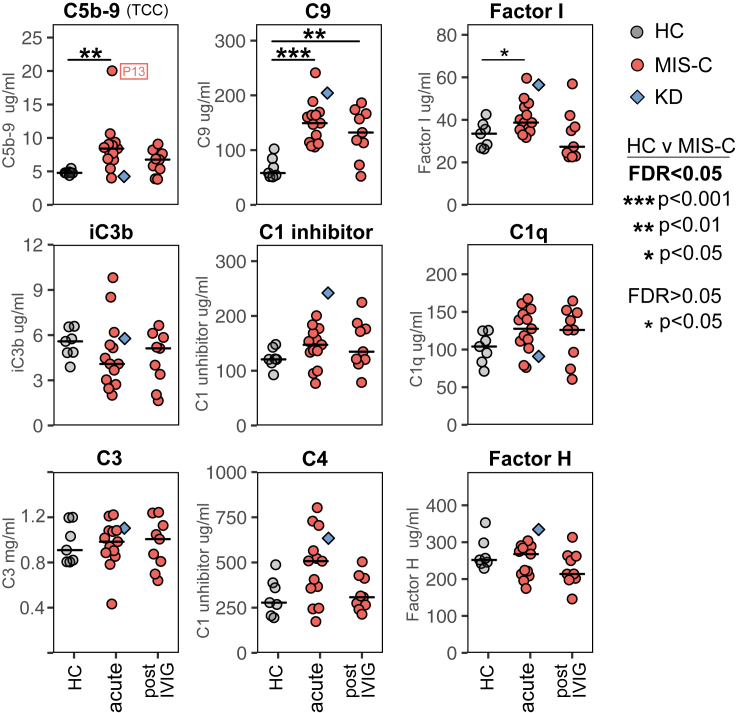
Analysis of complement in plasma samples from healthy children and patients Levels of the indicated complement components (C1q, C3, C4, C9), regulators (factor H, factor I) and activation products (iC3b, C5b-9/TCC) in plasma samples from seven healthy children (HC), thirteen MIS-C patients at the acute stage of disease and nine of these patients shortly after IVIG treatment. The horizontal line indicates the median value for patients and controls. Results of Wilcoxon rank-sum tests comparing the frequency of each cluster in healthy children to that in MIS-C patients at the acute or the post IVIG timepoints are indicated by: ∗p < 0.05, ∗∗p < 0.01, ∗∗∗p < 0.001. Non-significant results are not shown and emboldened p value symbols indicate significant results after 5% false discovery rate correction using the Benjamini-Hochberg method. Also shown on the plot, but not included in the statistical analysis, are the results from a single acute stage KD patient (blue diamond symbol, patient KD2). The level of C5b-9 for patient P13, who had severe disease, is indicated on the plot.

The complement system is a key part of the innate immune system and modulates adaptive immunity. Complement dysregulation has been repeatedly observed in COVID-19 (Holter et al., 2020; Magro et al., 2020; Zelek et al., 2020b). Quantification of nine complement markers (Figure 7A) revealed that complement protein C9 and C5b-9 (the terminal complement complex, TCC, and indicative of ongoing terminal pathway activation) were significantly increased in MIS-C patients at the acute stage of disease. The terminal pathway of the complement system generates membrane attack complex (MAC) pores that lyse cells but which also have potent pro-inflammatory action (Morgan, 2016). Notably, levels of C5b-9 were highest in MIS-C patient P13, who had the most severe disease, and lowest in KD patient KD2. The complement regulator Factor I was also raised at the acute stage. After treatment, C5b-9 levels decreased and were no longer significantly higher than controls.

## Discussion

MIS-C is a newly described and rare manifestation of SARS-CoV-2 infection of children that is clinically and temporally distinct to COVID-19 but shares features with several pediatric diseases including KD. Current treatment for MIS-C relies on supportive care (e.g., vasoactive medication) in combination with immunomodulatory agents. The most frequently used agent is IVIG (Ahmed et al., 2020; Hoste et al., 2021) which has been used to successfully treat KD for decades (Nagelkerke and Kuijpers, 2014). Although its mechanism of action is unknown IVIG acts rapidly, resolving inflammation in most KD patients within two days (Newburger et al., 1991). To investigate the immunopathology of MIS-C, and how this alters in response to therapy, we performed high dimensional immune analysis on blood samples taken from MIS-C and KD patients before treatment, 1–3 days after IVIG infusion when symptoms were resolving and upon ICU/hospital discharge.

The clinical features of our MIS-C cohort were consistent with other studies. Patients were older than 5 years old, had high blood levels of inflammatory markers, ferritnemia, neutrophilia, lymphopenia, and increased numbers of plasmablasts (Ahmed et al., 2020; Carter et al., 2020; Jiang et al., 2020; Vella et al., 2021; Hoste et al., 2021). Neutrophilia has previously been reported to correlate with IVIG resistance in KD (Kanai et al., 2020) and disease severity in adult COVID-19 patients (Wang et al., 2020). Our data revealed a significant positive correlation exists between increased neutrophil count and cardiac dysfunction, inflammation and disease severity in MIS-C. Because neutrophil count is a widely available test this observation provides a simple means for clinicians to assess disease severity and stratify patients in the future. Interestingly we also observed that all MIS-C patients in our study were deficient for vitamin D, which is linked to greater disease severity in KD (Stagi et al., 2016; Jun et al., 2017) and enhanced inflammation in general (Yin and Agrawal, 2014). In the UK vitamin D deficiency is common in the black and ethnic minority groups, which 15/16 of our MIS-C patients were from (Sutherland et al., 2020). We therefore suggest it is unlikely that vitamin D deficiency alone is responsible for the development of MIS-C but, given the wide-ranging effects of vitamin D on the immune system (Feketea et al., 2021), this finding warrants further investigation in larger MIS-C cohorts.

Our high dimensional analysis of acute MIS-C patients provides new insights into disease pathogenesis. Although in our study we were unable to directly compare MIS-C to COVID-19 cases, we were able to identify several new immune features shared between the two diseases. First, we found that neutrophilia correlates with disease severity in MIS-C, as is the case for COVID-19 in adults but not children (Guan et al., 2020). Neutrophils, which we and others have shown are highly activated in MIS-C, may be the source of the raised myeloperoxidase levels we detected in MIS-C patients' plasma. Further characterization of neutrophil effector molecules in MIS-C, particularly neutrophil elastase and neutrophil extracellular traps which have both been linked to KD and COVID-19 pathogenesis (Takeshita et al., 1999; Yoshida et al., 2020; Reusch et al., 2021) are clearly important priorities for future investigation. Second, we showed acute MIS-C patients had high plasma levels of pentraxin-3 (PTX3), an important innate immune mediator of resistance to viral infection and regulator of inflammation (Garlanda et al., 2018). PTX3 levels are raised in adult COVID-19 patients with plasma levels serving as a strong prognostic indicator of mortality (Brunetta et al., 2020). Endothelial cells are a major source of PTX3 and increased levels are reported for various vasculopathies. Third, we showed acute MIS-C patients had high levels of plasminogen activator inhibitor-1 (PAI-1), a marker of endothelial dysfunction that amplifies neutrophil-mediated inflammation via multiple mechanisms (Zmijewski et al., 2011; Ren et al., 2015) and that is raised in multiple inflammatory conditions including trauma, sepsis and in adult COVID-19 patients (Zuo et al., 2021). Fourth, we showed the complement pathway was activated in MIS-C, as demonstrated by high levels of C9 and C5b-9. The latter is a marker of activation of the membrane attack complex, the final common pathway of complement activation. MAC pore formation lyses cells and acts as a potent inflammatory trigger activating cytokine production by monocytes and neutrophils (Morgan, 2016). Complement activation can also cause endothelial damage and could explain the shock common in MIS-C patients. Plasma levels of C5b-9 are raised in adult COVID-19 patients with high levels being associated with severe disease and respiratory failure (Holter et al., 2020; Zelek et al., 2020b). Notably, all four of the above immune features that have previously been described in COVID-19, and which we identify here in MIS-C, have also been observed in previous studies of children with KD (Ching et al., 2020; Polycarpou et al., 2021). All four were also present in the KD patient we included in the high dimensional analyses, although we note their complement profile was distinct: high C9, low C5b-9 with high C1 inhibitor and factor H.

Analyzing MIS-C patients alongside a representative KD patient provided further insights into the two conditions. We acknowledge the limitation of analyzing a single KD patient but highlight that their immune profile was consistent with evidence from multiple studies of larger numbers of KD patients. These include: lymphopenia; neutrophilia; neutrophil activation; increased intermediate and non-classical monocytes; high CD64 expression on monocytes and neutrophils; and raised levels of IL-6, IL-18, PAI-1, sCD25, sTNF-R, IP-10, MPO, and PTX3 (Takeshita et al., 1999; Senzaki et al., 2003; Weng et al., 2013; Ko et al., 2015; Hokibara et al., 2016; Ching et al., 2020). Thus, they serve as a valuable exemplar of the disease in our analyses. MIS-C patients had all of the aforementioned features in common with KD with two notable exceptions. First, MIS-C patients possessed increased frequencies of DN1 B-cells, a subset unaltered in KD (Xu et al., 2019). DN1 B-cells are increased during pathogenic and protective immune responses and may represent precursor B-cells or early activated memory B-cells (Sanz et al., 2019; Ruschil et al., 2020). The second is that MIS-C patients lacked the expansion of intermediate and non-classical monocytes that is a hallmark of KD (Katayama et al., 2000; Hokibara et al., 2016). The function of these monocyte subsets, normally rare in the peripheral blood, is still being defined but they are generally considered to play a role in tissue repair (Olingy et al., 2017). Of particular relevance for KD vasculitis, the expression of CX3CR1 on NCM (Wong et al., 2011) and our scRNAseq data) allows them to closely interact with the vasculature (Auffray et al., 2007). Whether the lack of these cells in MIS-C is the reason why these patients present with severe shock, which is rare in KD, requires further investigation.

A key feature of our study was the analysis of paired samples taken from patients before treatment and then shortly afterwards during symptom resolution. This provides a unique window into pathogenesis and disease recovery. Shortly after treatment we observed rapid changes in both cytokine levels and immune cell phenotype. After only two days, levels of proinflammatory chemokines (IP-10 and MCP-1) and pro-inflammatory cytokines (IL-6, IL-18) had decreased. As noted by others, acute MIS-C patients also had elevated levels of the anti-inflammatory cytokine IL-10 and we show this also decreases after treatment. However, our data shows that the trajectory of recovery is more complex than a simple reversal of the hyper-inflammatory state. Thus, we observed substantial increases in plasma arginase levels in MIS-C patient 13 and KD patient 2 shortly after treatment. Although we did not identify the cellular source of arginase a prime candidate is neutrophils which were present at particularly high frequency in both patients. Arginase-positive immunosuppressive neutrophils have been detected in adult COVID-19 patients and other inflammatory conditions (Pillay et al., 2012; Tak et al., 2017; Schulte-Schrepping et al., 2020).

Analysis of paired samples also identified complex changes in cellular immunity occurring during recovery. Following treatment, classical monocytes expressed more CD163, a marker of anti-inflammatory monocytes. This could reflect previously activated monocytes transitioning to a less-activated state, no longer shedding CD163 because of downregulation of TACE, or the establishment of a new population of activated monocytes with anti-inflammatory activity (West et al., 2012). Neutrophils also changed during recovery. Thus, overall, levels of CD64 decreased but in parallel we also observed the emergence of a new population of unusual granulocytes (cluster 3) that expressed CD66b and CD63 but which lacked expression of CD35, CD55, and CD11c. Interestingly, these three proteins act as complement receptors or regulators. These unusual granulocytes were present at low frequency in healthy donors and acutely ill KD and MIS-C patients but their expression of CD64 and their frequency both increased during recovery, in one patient reaching up to 30% of total granulocytes.

All of the patients we studied by deep immune profiling received some kind of immunomodulatory therapy, either IVIG alone or IVIG in combination with steroids. Without an untreated control group for comparison we cannot definitively prove that the immunological changes we saw during recovery were treatment induced: they could be a natural process of recovery from the acute inflammatory state of MIS-C. However, assuming they were iatrogenic in origin then the fact that they occurred in patients that received IVIG alone suggests this agent was the cause. Our study therefore reveals potential new modes of action for IVIG for validation in larger studies of MIS-C, KD or other inflammatory conditions.

A key aim of our study was to provide a rational basis for MIS-C therapy, which to date has empirically followed protocols used to treat KD. These include IVIG, steroids and targeted agents that selectively inhibit IL-6 (Tocilizumab), IL-1β (Anakinra) or TNF-α (Infliximab) (Ahmed et al., 2020; Jiang et al., 2020; Hoste et al., 2021). The consistent and rapid immune changes that occurred in our MIS-C and KD patients shortly after IVIG (including patients who received this drug as a single agent) are striking, supporting the continued use of IVIG in MIS-C. Cases that fail to respond to these agents may need additional therapies. Our results are highly relevant for guiding treatment choices in such cases. First, we showed MIS-C patients had much higher levels of IL-6 relative to IL-1β and TNF-α in plasma. Furthermore, MIS-C patients also had high levels of IL1-RA (the natural IL1 receptor antagonist protein, present in plasma but also produced in recombinant form as the drug Anakinra) and soluble TNF receptors (which inhibit TNF-α signaling *in vitro* (Balcewicz-Sablinska et al., 1998)). This combination of low levels of IL-1β and TNF-α but high levels of their natural antagonists suggests additional therapeutic inhibition of these pathways may have limited benefit. Taken together, these observations suggest IL-6 inhibition may be the preferred choice for anti-cytokine therapy. Second, our data strongly suggest that complement inhibition, with appropriate prophylactic antibiotic cover, should be considered as a therapeutic approach. Of the 13 MIS-C patients we analyzed, 9 (69%) had plasma C5b-9 levels greater than 7.14ug/ml, the cut-off value used to stratify adult severe COVID-19 patients for complement inhibitor LFG316 therapy in a recent compassionate use study (Zelek et al., 2020b). Our data therefore provides a rational basis for testing inhibitors of C5b-9 (such as LFG316 or eculizumab, the latter already licensed for use in children with atypical hemolytic uremic syndrome or thrombotic microangiopathy) (Fakhouri et al., 2016) or inhibitors of upstream complement activation pathways (Polycarpou et al., 2021) in clinical trials.

### Limitations of the study

This was a non-randomized single center observational study performed under emergency pandemic conditions investigating a rare, newly described disease. Because observational studies cannot prove causation we cannot exclude the possibility that the immunological changes we observed over time may not be treatment related. Observational studies are sensitive to confounders and selection bias but provide a better estimate of clinical practice. The potentially confounding effects of patient heterogeneity was reduced in our study by ensuring all study patients had serological evidence of prior SARS-CoV-2 infection. Experimental variability was controlled as follows. First, our pretreatment and posttreatment samples were obtained from the same individuals, eliminating any inter-individual heterogeneity in the longitudinal analysis. Second, all patient and control samples were processed in a single laboratory by one of two individuals who followed a standardized protocol. Third, all study samples were analyzed at the same time in single scRNAseq, mass cytometry, cytokine profiling and complement analysis experiments, eliminating inter-assay variance. The number of patients and number of cells analyzed by scRNAseq was low. Therefore, these data were primarily treated as a discovery set, shaping the design of the mass cytometry panel which was then applied to a larger number of samples including those analyzed by scRNAseq. Finally, although the number of patients we recruited is small it is comparable to most other MIS-C studies. We highlight that all but one of our acute phase MIS-C samples were collected from patients before treatment commenced (the exception being P14, with their first sample being collected eight hours after IVIG infusion). Our data therefore provide a more accurate assessment of acute MIS-C than other studies that have relied entirely upon samples collected days after immune modulating treatments had been administered to patients.

## STAR★Methods

### Key resources table

**Table:** 

REAGENT or RESOURCE	SOURCE	IDENTIFIER
**Antibodies**		

Totalseq-B Human TBNK panel	Biolegend	Cat#399902
Totalseq-B anti-human TCR g/d	Biolegend	Cat#331233
Totalseq-B anti-human CD45RO	Biolegend	Cat#304257
Anti-CD41 / CD61 (clone A2A9/6,purified)	Biolegend	Cat#359802
Anti-CD42a (clone REA209, purified)	Miltenyi	Cat#130-122-338
Anti-CD16 (clone 3G8, purified)	Biolegend	Cat#302002
Anti-CD14 (clone RMO52, purified)	Beckman Coulter	Cat#IM0643
Anti-CD2 (clone TS1/8, purified)	Biolegend	Cat#309219
Anti-CD8 (clone SK1, purified)	Biolegend	Cat#344727
Anti-CD57 (clone HCD57, purified)	Biolegend	Cat#359602
Anti-CD36 (clone 5-271, purified)	Biolegend	Cat#336202
Anti-FceR1 (clone AER-37, purified)	Biolegend	Cat#334602
Anti-CD45 (clone HI30, purified)	Biolegend	Cat#304002
Anti-CD19 (clone HIB19, purified)	Biolegend	Cat#302247
Anti-CD32 (clone FUN-2, purified)	Biolegend	Cat#303202
Anti-CD4 (clone RPA-T4, conjugated to 145Nd)	Fluidigm	Cat#3145001B
Anti-IgD (clone IA6-2, conjugated to 146Nd)	Fluidigm	Cat#3146005B
Anti-CD11c (clone S-HCL-3, purified)	Biolegend	Cat#371502
Anti-CD69 (clone REA824, purified)	Miltenyi	Cat#130-124-326
Anti-CD64 (clone 10.1, purified)	Biolegend	Cat#305029
Anti-CD62L (clone DREG56, purified)	Biolegend	Cat#304802
Anti-CD123 (clone 6H6, purified)	Biolegend	Cat#306002
Anti-CD45RA (clone HI100, purified)	Biolegend	Cat#304102
Anti-CD177 (clone MEM-166, purified)	Biolegend	Cat#315802
Anti-CD86 (clone IT2.2, purified)	Biolegend	Cat#305402
Anti-CD39 (clone A1, purified)	Biolegend	Cat#328202
Anti-CD163 (clone GHI/61, purified)	Biolegend	Cat#333602
Anti-CD55 (clone JS11, purified)	Biolegend	Cat#311302
Anti-CD56 (clone NCAM16.2, conjugated to 163Dy)	Fluidigm	Cat#3163007B
Anti-CD95 (clone DX2, purified)	Biolegend	Cat#305602
Anti-CD35 ((clone E11, purified)	Biolegend	Cat#333402
Anti-CD27 (clone L128 conjugated to 167Er)	Fluidigm	Cat#3167006B
Anti-CD10 (clone HI10a, purified)	Biolegend	Cat#312202
Anti-CD25 (clone 2A3 conjugated to 169Tm)	Fluidigm	Cat#3169003B
Anti-CD3 ((clone UCHT1, purified)	Biolegend	Cat#300402
Anti-CD40 (clone HB14, purified)	Biolegend	Cat#313002
Anti-CXCR4 (clone 12G5 conjugated to 175Lu)	Fluidigm	Cat#3175001B
Anti-CD63 (clone H5C6, purified)	Biolegend	Cat#353039
Anti-CD235 (clone HI264, purified)	Biolegend	Cat#349102
Anti-CD38 (clone HIT2, purified)	Biolegend	Cat#303502
Anti-HLA-DR (clone L243, purified)	Biolegend	Cat#307602

**Biological samples**		

Whole Blood, PBMC, serum and plasma from MIS-C patients, KD patients and healthy children.	N/A	N/A

**Chemicals, peptides, and recombinant proteins**		

Stabilised trimeric SARS-CoV2 Spike protein	The Binding Site	Cat#MK654
Stabilcoat	Sigma Aldrich	Cat#S0950
Sheep anti-human IgG HRP	The Binding Site	Cat#MK654
Sheep anti-human IgM HRP	The Binding Site	Cat#MK654
Sheep anti-human IgA HRP	The Binding Site	Cat#MK654
TMB	The Binding Site	Cat#MK654
Orthophosphoric acid	The Binding Site	Cat#MK654
Cytodelics whole blood processing kit	Cytodelics ab	Cat#hC002-1000
Maxpar X8 Antibody Labelling kits (various isotopes)	Fluidigm	various
Maxpar MCP9 Antibody Labelling kits (various isotopes)	Fluidigm	various
EQ four element calibration beads	Fluidigm	Cat#201078
Trustain Fc receptor blocking solution	Biolegend	Cat#422302
Cell-ID iridium intercalator	Fluidigm	Cat#201192B
Cell Staining Media (CSM, phosphate buffered saline + 0.5% foetal bovine serum + 0.02% sodium azide)	Prepared in house	N/A
Nunc MaxiSorp Plates	Thermo Fisher	Cat#44-2404-21
Tween 20	VWR	Cat#663684B
Bovine Serum Albumin	Thermo Fisher	Cat#10257123
Sigmafast OPD	Sigma Aldrich	Cat#P9187
TMB	Thermo Fisher	Cat#555214
Mouse anti-TCC (aE11)	Hycult	Cat#HM2167-1MG
Mouse anti-TCC (E2 conjugated to biotin)	E2: in-house Biotin: Thermo Fisher	Cat#21327
Mouse anti-C9 (B7)	In house	
Polyclonal rabbit anti-C9 (conjugated to HRP)	In house	
Mouse anti-factor I (7B5)	In house	
Polyclonal rabbit anti-factor I	In house	
Mouse anti-C1q (9H10)	In house	
Polyclonal rabbit anti-C1q	In house	
Mouse anti-factor H (Ox-24)	In house	
Mouse anti-factor H (35H9)	In house	
Mouse anti-iC3b (clone 9)	In house	
Mouse anti-iC3b (bH6, conjugated to HRP in house)	Hycult	Cat#HM2168
Mouse anti-C1 inhibitor	In house	
Polyclonal rabbit anti-C1 inhibitor	In house	
Polyclonal rabbit anti-C4	In house	
Polyclonal rabbit anti-C4 (conjugated to HRP)	In house	
Polyclonal rabbit anti-C3	In house	
Polyclonal rabbit anti-C3 (conjugated to HRP)	In house	
EZlink HRP labelling kit	Thermo Fisher	Cat#31489
Donkey anti-rabbit IgG HRP	Jackson ImmunoResearch	Cat#711-035-152
Goat anti-human IgM HRP	Thermo Fisher	Cat#A18841
Streptavidin HRP	Thermo Fisher	Cat#21130
TCC protein	Purified in house	
C9 protein	Purified in house	
Factor I protein	Purified in house	
C1q protein	Purified in house	
C3 protein	CompTech	Cat#A113
C4 protein	Purified in house	
Factor H protein	Purified in house	
iC3b protein	CompTech	Cat#A115
C1 inhibitor protein	Purified in house	

**Critical commercial assays**		

BD Multitest trucount tubes (6 colour)	Beckton Dickinson	Cat#337166
Legendplex macrophage.microglia 13-plex panel	Biolegend	Cat#740502
Legendplex Human Inflammation panel 1	Biolegend	Cat#740808
Legendplex Inflammation panel 2	Biolegend	Cat#740775
Myeloperoxidase ELISA	Thermo Fisher	Cat#BMS2038INST
Chromium Next GEM Single cell 3’ GEM, Library and Gel Bead kit v3.1	10x Genomics	Cat#1000128
Chromium Next GEM Chip G Single Cell Kit	10x Genomics	Cat#1000127
Chromium i7 Multiplex Kit	10x Genomics	Cat#220103
Chromium Single Cell 3' Feature Barcode Library Kit	10x Genomics	Cat#1000079
Illumina NextSeq High 150 v2.5	Illumina	Cat#20024907
Illumina NextSeq Mid 150 v2.5	Illumina	Cat#20024904

**Deposited data**		

Single cell RNA-seq data (deposited to GEO)	www.ncbi.nlm.nih.gov/geo/	GSE183716

**Software and algorithms**		

R (version 4.0.3)	https://cran.r-project.org	N/A
CellRanger	10X Genomics	N/A
Seurat	https://cran.r-project.org/web/packages/Seurat	N/A
Revigo	http://revigo.irb.hr	N/A
Prism	GraphPad	N/A
Legendplex Cytokine Analysis Software	Biolegend	N/A
Cytobank Cytometry Analysis Software	Cytobank	N/A

**Other**		

Helios Mass Cytometer	Fluidigm	N/A
LSRFortessa X-20 Cytometer	Beckton Dickinson	N/A
FACSCanto-II Cytometer	Beckton Dickinson	N/A

### Resource availability

#### Lead contact

Further information and any requests should be directed to and will be fulfilled by the lead contact, Graham Taylor (g.s.taylor@bham.ac.uk).

#### Materials availability

This study did not generate new unique reagents.

### Experimental model and subject details

#### Ethical approvals and patient demographic information

All patient samples were obtained Birmingham Children's Hospital as part of a Health Research Authority approved study (TrICICL) reviewed and approved by South of Birmingham Research Ethics Committee (REC: 17/WM/0453, IRAS: 233593). Demographic and clinical information for each patient is provided in Table S1. Samples from seven healthy children (aged 12 years) were obtained via the Coronavirus Immunological Analysis study approved by North West - Preston Research Ethics Committee (REC: 20/NW/0240, IRAS: 282164). This study was performed in accordance with the declaration of Helsinki and written informed consent was obtained from all participants or their legal guardians.

### Method details

#### Sample processing and storage

Peripheral blood samples from healthy donors or paediatric patients presenting with suspected MIS-C or KD were collected in EDTA and serum vacutainer tubes and stored at room temperature before processing. Whole blood was preserved using Cytodelics stabiliser and peripheral blood mononuclear cells (PBMCs) isolated using SepMate tubes as per the manufacturer’s protocol and cryopreserved in a solution of 70% fetal calf serum and 10% dimethyl sulfoxide using an optimised protocol (Kreher et al., 2003). Plasma collected during PBMC isolation and serum separated by centrifugation were stored as frozen aliquots at -80°C.

#### Clinical laboratory data

Antibodies specific for the SARS-CoV-2 spike protein were detected by ELISA (Perez-Toledo et al., 2020). ELISA plates coated with stabilized, trimeric spike glycoprotein truncated at the transmembrane region (The Binding Site, UK) were blocked with Stabilcoat solution and serum samples pre-diluted at a 1:40 dilution using a Dynex Revelation automated liquid handler (Dynex, USA) were added. Bound antibodies were detected using sheep-anti-human horseradish peroxidase (HRP)-conjugated polyclonal antibodies against IgG (1:16,000), IgA (1:2000), and IgM (1:8000), TMB core and orthophosphoric acid as a stop solution (all from The Binding Site, UK). Optical densities at 450nm were measured using the Dynex Revelation automated liquid handler. IgG, IgA, and IgM ratio-cutoffs were determined based on running 90 pre-2019 negative serum samples. Ratio values > 1, are classed as positive and ratio values < 1 are classed as negative for anti-SARS-CoV-2 spike IgG, IgA or IgM antibodies. Clinical laboratory tests were performed at the Birmingham Children’s Hospital’s clinical laboratory. Lymphocyte subset enumeration was performed by the University of Birmingham Clinical Immunology Service using Trucount tubes and a FACSCanto-II cytometer (Key resources table). Paediatric reference values were obtained from previously published data (Comans-Bitter et al., 1997).

#### scRNA sequencing

All blood samples used for single cell RNA-seq were cryopreserved within four hours of phlebotomy. Cryopreserved PBMCs were recovered, feature barcoded using DNA-conjugated antibodies and libraries constructed using 10X Genomics reagents (Key resources table) according to manufacturer’s instructions. Sequencing was performed on an Illumina NextSeq 500 platform, sequencing 419 million reads of 150 bases. All samples were recovered, processed and sequenced at the same time.

#### Mass cytometry

Antibodies were purchased pre-conjugated from Fluidigm or unconjugated from other suppliers and conjugated in house using Fluidigm Maxpar reagents (Key resources table and Table S2). Whole blood samples stored in Cytodelics stabiliser media were thawed and processed as per manufacturer's protocol. For staining, a master-mix of all 38 phenotyping antibodies was prepared by adding the appropriate pre-tested dilutions into filtered cell stain media (CSM – Key resources table). The antibody cocktail was then filtered through a 100 μm spin column before use. Cells were incubated with Fc block for 10 minutes, antibodies were added and incubated for further 30 minutes. The samples were washed twice with CSM then fixed overnight with freshly prepared 1.6% formaldehyde. The following day, cells were incubated with iridium intercalator solution for one hour then analysed on a Helios mass cytometer using an acquisition rate below 500 events per second. Immediately prior to acquisition cells were washed once in CSM buffer and twice in deionised water. Prior to acquisition, each sample was reconstituted in deionised water spiked with EQ calibration beads and filtered through a 70μm cell strainer.

#### Cytokine and complement quantification

Cytokines were quantified in plasma samples using three BioLegend LEGENDplex cytokine detection assays performed in parallel Assay beads were measured using an LSRFortessa X-20 cytometer. Myeloperoxidase was measured in plasma samples by ELISA. Complement proteins, regulators and activation markers were measured using established in-house ELISAs (Kopczynska et al., 2018; Zelek et al., 2020a). Nunc MaxiSorp plates were coated with capture antibody at 4⁰C overnight, then blocked (1h 37⁰C) with 2% bovine serum albumin (BSA) in phosphate buffered saline + 0.05% Tween-20 (PBS-T). After washing with PBS-T, protein standards or serum samples diluted in 0.2% BSA in PBS-T were added to plates in duplicate for 90 minutes at 37⁰C. Plates were washed 3x with PBS-T, then incubated with detection antibody for 1h (depending on the assay, some detection antibodies were labelled with HRP). For assays using unlabelled detection reagents plates were washed 3x with PBS-T then incubated with HRP-conjugated secondary antibody as above. After washing 3x with PBS-T assays were developed using either o-phenylenediamine dihydrochloride or 3,3',5,5'-tetramethylbenzidine and stopped with 5% sulphuric acid (see Key resources table for full details)

### Quantification and statistical analysis

#### ScRNAseq data pre-processing and quality control

Processing of raw reads including 10X barcode-aware demultiplexing from BCL to FASTQ files, transcriptome alignment to human genome assembly GRCh38 and unique molecular identifier (UMI) counting were performed using the 10X Cell Ranger pipeline version 3.1.0 with GRCh38-version 3.0.0 as the reference. Seurat v3.1.5 (Butler et al., 2018) was used for sample merging, quality control (QC), clustering and reporting. Both gene expression (RNA) and antibody- derived tag (ADT) assays were loaded from the CellRanger count platform into a Seurat object for each sample excluding cells with less than 200 genes and features detected in less than 3 cells. The four samples were then merged to create a single aggregated object. As application of batch effect correction (data integration) did not affect our conclusions we decided that batch effects were not a concern and chose to present the unaltered data. QC was conducted on both assays separately. For the RNA assay, to mitigate the influence of sex on clustering and differential expression, the XIST and RSP4Y1 genes were removed. Cells with a number of features between 200 and 6000, counts of greater than 1000 and mitochondrial percentage less than 15% were kept for further processing. The gene expression data was normalised using the ‘LogNormalize’ method with the scale.factor set to the default 10000. 1500 variable features were identified using the ‘vst’ method of the ‘FindVariableFeatures’ function. The assay was finally scaled with the number of counts and mitochondrial percentage variables regressed. The ADT assay was first used to identify dead cells or cell doublets by removing double positive instances of lineage markers according to the following criteria: CD14 > 60 & CD19 > 50, CD19 > 40 & CD3 > 40, CD14 > 80 & CD3 > 50 and CD3 < 50 & gamma-delta > 400. The assay was then centred log-ratio (CLR) normalised and scaled with default parameters. The remaining object comprised 10,031 cells with 17,583 features.

#### PBMC clustering of scRNAseq data

Both the RNA and ADT assays were used to compute 50 principal components. These principal components were then used as input into the ‘FindNeighbours’ function and subsequently the ‘FindClusters’ function with resolution set to the default value of 1, resulting in 19 clusters which were visualised using tSNE. The clusters were manually annotated from expression of lineage markers both at the transcript (RNA) and protein (ADT) levels. To check our samples for potential T-cell activation we evaluated our data for expression of genes previously identified by scRNAseq analysis as being upregulated upon T-cell stimulation (Szabo et al., 2019).

#### Monocyte clustering of scRNAseq data

To examine the monocyte populations in finer detail, we extracted clusters 4, 6, 9, 10 and 13 from Figure 1E using the ‘Subset’ function. As monocytes were abundant within each sample, all monocyte clusters were included (2248 monocytes extracted). Both assays were normalized separately (RNA: ‘LogNormalize’ & ADT: ‘CLR’). The ‘vst’ method was used to find the 2000 most variable features within the RNA assay and the data was scaled with the variables ‘number of counts’ and ‘percent mitochondria’ regressed. The ADT assay was scaled with default parameters. In this instance, 100 principal components were used in the ‘RunPCA’ function with the top 50 of those components used to find cell neighbours and clusters. The UMAP reduction was selected here as the clusters visually displayed a better path from classical to intermediate to non-classical monocytes over the tSNE reduction. Nine clusters were originally generated with further assessment resulting in the identification of seven monocyte clusters. CD14 and CD16 markers from the ADT assay were used to classify classical (CD14^+^), non-classical (CD16^+^) and intermediate (CD14^+^CD16^+^) monocyte populations. Further differential expression analysis of the RNA assay was used to define cellular function within each cluster.

#### Gene ontology

Differential gene expression was conducted between the acute samples and the convalescent sample for CD14^+^ monocytes, NK, CD8^+^ T cells, CD4^+^ T cells and B cells with the ‘FindMarkers’ function in Seurat using MAST (Finak et al., 2015). Both up and down regulated genes were identified. Gene ontology was conducted with the resulting gene lists using EnrichR (Chen et al., 2013), focusing on the biological processes subset (‘GO_Biological_Processes’). Go terms were simplified using Revigo software (Supek et al., 2011).

#### Mass cytometry

Mass cytometry data was normalised using the CyTOF data acquisition software (Fluidigm). Normalised data were exported as FCS (Flow Cytometry Standard) files and uploaded to Cytobank for doublet discrimination and analysis by manual gating and unsupervised methods. Singlet cells from healthy children or MIS-C patients at different clinical stages were examined individually or as concatenated FCS files comprising an equal number of cells from each individual. Each FCS file was then downsampled to 16,000 cells (mononuclear cells) or 22,000 cells (granulocytes) for analysis. Gating strategies are provided in Figure S11. Dimensionality reduction and clustering were performed in Cytobank using the ViSNE implementation of tSNE and FlowSOM respectively.

#### Legendplex cytokine measurement

Data were analysed using the LEGENDplex data analysis software provided by the manufacturer, following their recommendations.

#### Data analysis and statistical testing

Time series of positive cases by testing date for England was downloaded from the UK Government Department of Social Care and Public Health England website: https://www.gov.uk/guidance/coronavirus-covid-19-information-for-the-public#time-series-documents. Data shown are results for area ‘Birmingham’. Data analysis was performed in R version 4.0.3 using the lm, marixStats and matrixTests packages and figures prepared using ggplot2. Box and whisker plots show the standard Tukey representation (median, interquartile range and whiskers extending to the largest and smallest values within 1.5 times the interquartile range with outliers plotted as points). We report the significance of each test and, where necessary, correct for multiple comparisons by controlling the false discovery rate (FDR) using the Benjamini-Hochberg procedure with a stringent FDR of 5%. The details of each test are provided in figure legends. When analysing patients samples collected at different timepoints (acute, post IVIG and discharge) we compared values from each timepoint to the healthy donor values. Principal component analysis was performed using the PCAtools package in R. For the PCA analysis of clinical laboratory data we did not have access to data from healthy individuals but normal reference values were available. Therefore, to allow patients to be compared to the reference values (some of which vary by age or gender, Figure 1) we generated synthetic healthy controls. For each patient we generated 10 synthetic controls by randomly selecting values for each feature within the normal range that would be expected for that patient based on their age and gender. Following PCA the synthetic healthy donors therefore indicate where healthy donors would localise in the lower dimensionality projection. For cytokines (Figure 7) the PCA plot was prepared using the 24 features with highest variance (variance > 300). PCA analyses were performed using scaled and centred values. Graphs were prepared using ggplot2 or Prism software version 8. The correlation matrix (Figure 1) was produced in using publicly available code downloaded from GitHub on the 3^rd^ November 2020 (Wherry research group, University of Pennsylvania). Any additional information required to reanalyse the data reported in this paper is available from the lead contact upon request.

## Data Availability

Raw and processed single cell RNA-seq data have been deposited online in GEO and are publicly available (www.ncbi.nlm.nih.gov/geo/ accession number GSE183716).

## Footnotes

Supplemental information can be found online at https://doi.org/10.1016/j.isci.2021.103215.
