# Supplementary material for: The immune landscape of SARS-CoV-2-associated Multisystem Inflammatory Syndrome in Children (MIS-C) from acute disease to recovery
Source: iScience. 2021 Oct 2;24(11):103215. doi: 10.1016/j.isci.2021.103215 (PMC8487319; doi:10.1016/j.isci.2021.103215)

iScience, Volume *24*

Supplemental information

The immune landscape of SARS-CoV-2-associated Multisystem Inflammatory Syndrome in Children (MIS-C) from acute disease to recovery

Eleni Syrimi, Eanna Fennell, Alex Richter, Pavle Vrljicak, Richard Stark, Sascha Ott, Paul G. Murray, Eslam Al-Abadi, Ashish Chikermane, Pamela Dawson, Scott Hackett, Deepthi Jyothish, Hari Krishnan Kanthimathinathan, Sean Monaghan, Prasad Nagakumar, Barnaby R. Scholefield, Steven Welch, Naeem Khan, Sian Faustini, Kate Davies, Wioleta M. Zelek, Pamela Kearns, and Graham S. Taylor

Supplementary Figures and Tables

Table S1. Demographic and clinical data for the 16 patients with MIS-C and 2 patients with Kawasaki disease (KD) recruited to the study. Related to Figure 1. KD patients are indicated by grey background. PCR: polymerase chain reaction. D=Diarrhoea, V=Vomiting, Neg= Negative, Pos= Positive, M=Male, F=Female. Cardiac involvement includes abnormal ECG and/or echocardiography findings.

ID	Age (years)	Sex	Ethnicity	Fever	Rash	Lymph-adenopathy	Conjunctivitis non-exudated	Mucosal changes	Peripheral changes	GI features	Cardiac involvement	PCR for SARS-CoV-2	Serology for SARS-CoV-2	Diagnosis
1	0-4	M	Asian British	Yes	Yes	No	No	No	No	No	NO	Neg x3	Not done	KD
2	0-4	M	White British	Yes	Yes	Yes	Yes	Yes	Yes	Yes, V	NO	Neg x1	Negative	KD
3	5-10	M	Black British-African	Yes	No	No	No	No	No	Yes, V	YES	Neg x3	Positive	MIS-C
4	5-10	M	Asian British-Pakistani	Yes	Yes	No	Yes	Yes	Yes	Yes, abdominal pain, V	NO	Neg x1	Positive	MIS-C
5	5-10	M	Asian British-Pakistani	Yes	Yes	Yes	Yes	Yes	No	Yes, acute abdomen, pain, V	Yes	Neg x3	Positive	MIS-C
6	5-10	F	Black British	Yes	Yes	No	Yes	Yes	No	Yes, amdominal pain, D	Yes	Pos x1 Neg x2	Positive	MIS-C
7	5-10	F	Asian British-Indian	Yes	Yes	No	No	No	No	Yes, abdominal pain, V	NO	Pos x2	Positive	MIS-C
8	5-10	M	Asian British-Indian	Yes	No	No	No	No	No	Yes, acute abdomen, pain, D&V	YES	Neg x3	Positive	MIS-C
9	5-10	F	Asian British-Pakistani	Yes	Yes	No	No	No	No	Yes, pain, D&V	YES	Negx2	Positive	MIS-C
10	5-10	F	Black British-African	Yes	Yes	No	Yes	Yes	Yes	Yes,D&V	YES	Neg x2	Positive	MIS-C
11	5-10	M	Black British-Carribean	Yes	Yes	No	Yes	Yes	Yes	Yes, abdominal pain, D&V	YES	Neg x2	Positive	MIS-C
12	5-10	M	Asian British-Baghladeshi	Yes	No	No	Yes	No	Yes	Yes, abdominal pain, D&V	YES	Neg x2	Positive	MIS-C
13	5-10	F	Black British-African	Yes	Yes	Yes	No	Yes	No	Yes, acute abdomen, D&V	YES	Neg x2	Positive	MIS-C
14	11-15	F	Mixed White Black-Caribbean	Yes	No	No	No	No	No	Yes, admominal pain, D	YES	Neg x3	Positive	MIS-C
15	11-15	M	Mixed White Black- Afro Caribbean	Yes	No	No	No	No	No	Yes, abdominal pain, D&V	YES	Neg x2	Positive	MIS-C
16	11-15	F	White-Romanian	Yes	Yes	Yes	Yes	No	No	Yes, abdominal pain, V	YES	Neg x2	Positive	MIS-C
17	11-15	F	Black British-Caribbean	Yes	Yes	No	No	No	No	Yes, D&V	YES	Neg x3	Positive	MIS-C
18	11-15	M	Asian British- [illegible]	Yes	Yes	No	Yes	No	No	Yes, [illegible]	NO	Neg x3	Positive	MIS-C

RCPCH 2020 guidelines for PIMS-TS: A child presenting with persistent fever, inflammation (neutrophilia, elevated CRP and lymphopenia) and evidence of single or multi-organ dysfunction (shock, cardiac, respiratory, renal, gastrointestinal or neurological disorder). This may include children meeting full or partial criteria for Kawasaki disease. Exclusion of any other microbial cause, including bacterial sepsis, staphylococcal or streptococcal shock syndromes, infections associated with myocarditis such as enterovirus (waiting for results of these investigations should not delay seeking expert advice). SARS-CoV-2 PCR testing may be positive or negative

Table S2. **Antibody Markers used for Mass cytometry.** Related to Figures 3,4,5.

Metal	Marker	Clone	Source
89Y	CD41/42a61	A2A9/6 & REA209	Biolegend & Miltenyi
106Cd	CD16	3G8	Biolegend
110Cd	CD14	RMO52	Beckman Coulter
113Cd	CD2	TS1/8	Biolegend
114Cd	CD8	SK1	Biolegend
115In	CD57	HCD57	Biolegend
116Cd	CD36	5-271	Biolegend
139La	FCeR1	AER-37	Biolegend
141Pr	CD45	H130	Fluidigm
142Nd	CD19	HIB19	Fluidigm
144Nd	CD32	FUN-2	Biolegend
145Nd	CD4	RPA-T4	Fluidigm
146Nd	IgD	IA6-2	Fluidigm
147Sm	CD11c	5-HCL-3	Biolegend
148Nd	CD69	REA824	Miltenyi
149Sm	CD64	10.1	Biolegend
150Nd	CD62L	DREG56	Biolegend
151Eu	CD123	6H6	Biolegend
155Gd	CD45RA	HI100	Fluidigm
156Gd	CD177	MEM-166	Biolegend
159Tb	CD86	IT2.2	Biolegend
160Gd	CD39	A1	Fluidigm
161Dy	CD163	GHI/61	Biolegend
162Dy	CD55	IS11	Biolegend
163Dy	CD56	NCAM16.2	Fluidigm
164Dy	CD95	DX2	Biolegend
166Er	CD35	E11	Biolegend
167Er	CD27	L128	Fluidigm
168Er	CD10	H10a	Biolegend
169Tm	CD25	2A3	Fluidigm
173Yb	CD3	UCHT1	Biolegend
174Yb	CD40	HB14	Biolegend
175Lu	CXCR4	12G5	Fluidigm
176Yb	CD63	H5C6	Biolegend
194Pt	CD66b	6/40c	Biolegend
195Pt	CD235	HI264	Biolegend
196Pt	CD38	HIT2	Biolegend
198Pt	HLA-DR	L243	Biolegend

Figure S1. Time-course of monocyte, lymphocyte, granulocyte absolute counts and serum C-reactive protein levels over time. Related to Figure 1. Treatment is indicated by the arrows on each graph as follows: ivig= Intravenous Immunoglobulin, S= IV Steroids, Toc=Tocilizumab (anti-IL-6).

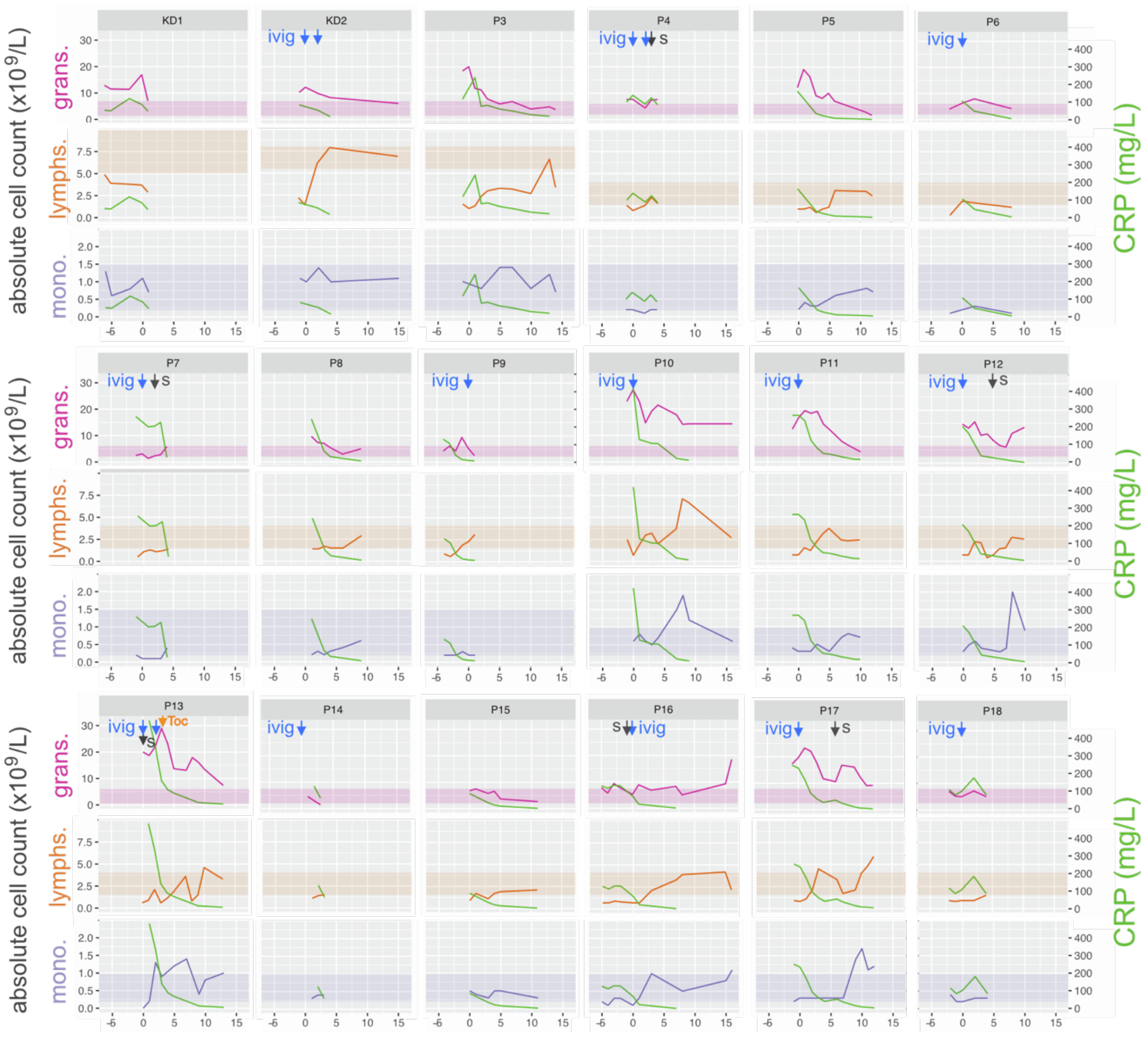

Figure S2. Gene expression profile of 19 clusters generated from patient PBMC samples. Related to Figure 2.

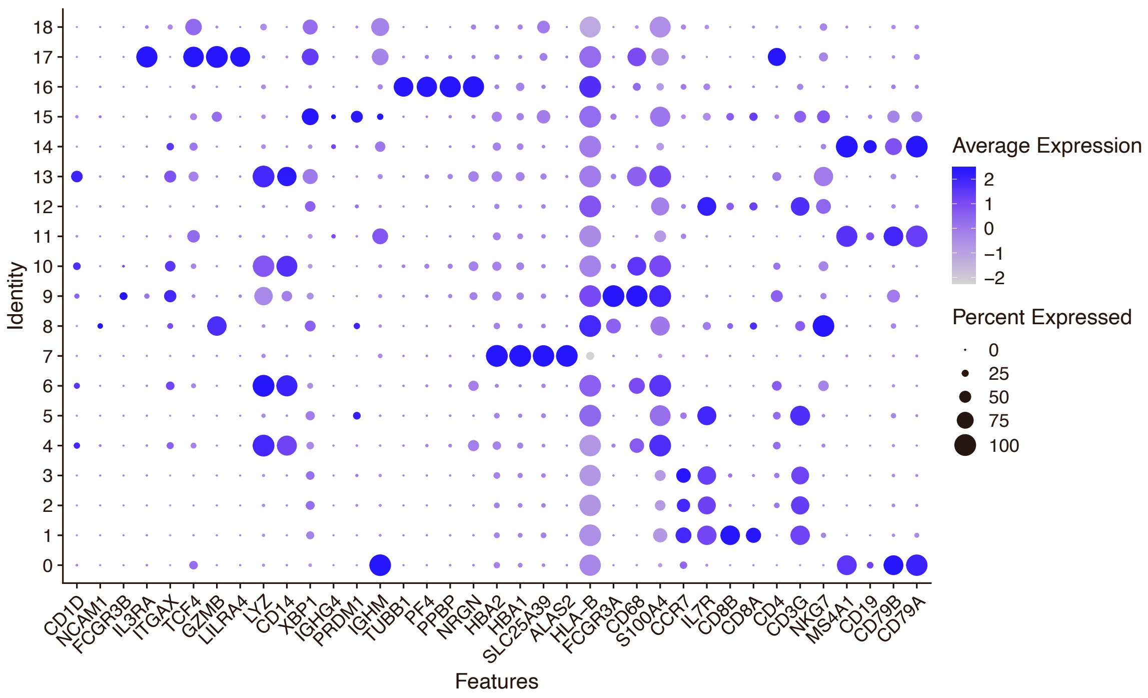

Figure S3. Heatmap showing scaled expression of discriminative genes sets for each PBMC cluster. Related to Figure 2. Only the top five discriminative genes per cluster are shown.

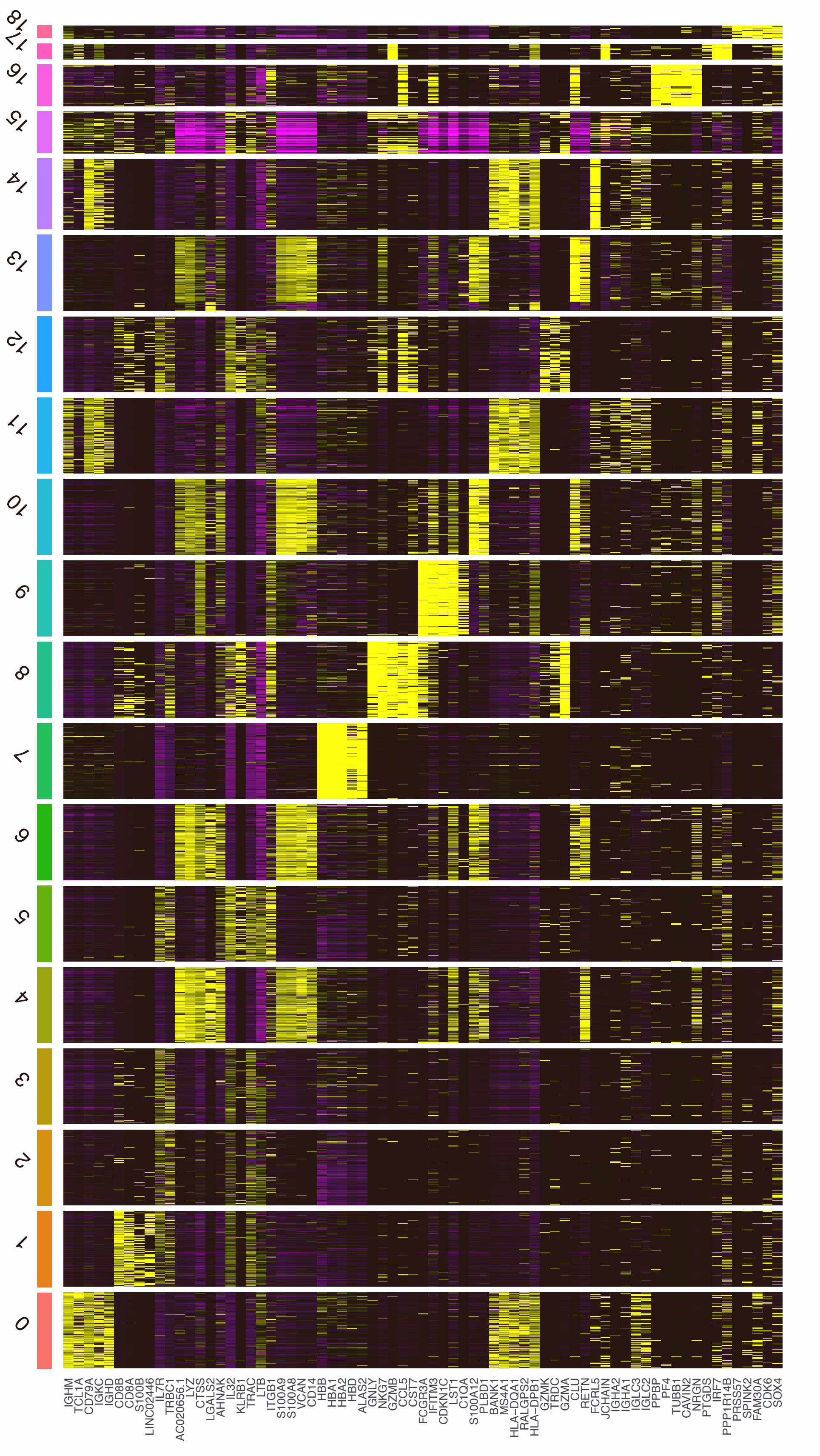

Figure S4. RNA expression and protein levels in patient PBMCs. Related to Figure 2. tSNE representation of combined PBMCs from all four patient samples coloured by the level of RNA per cell or level of protein per cell measured using antibody derived tags.

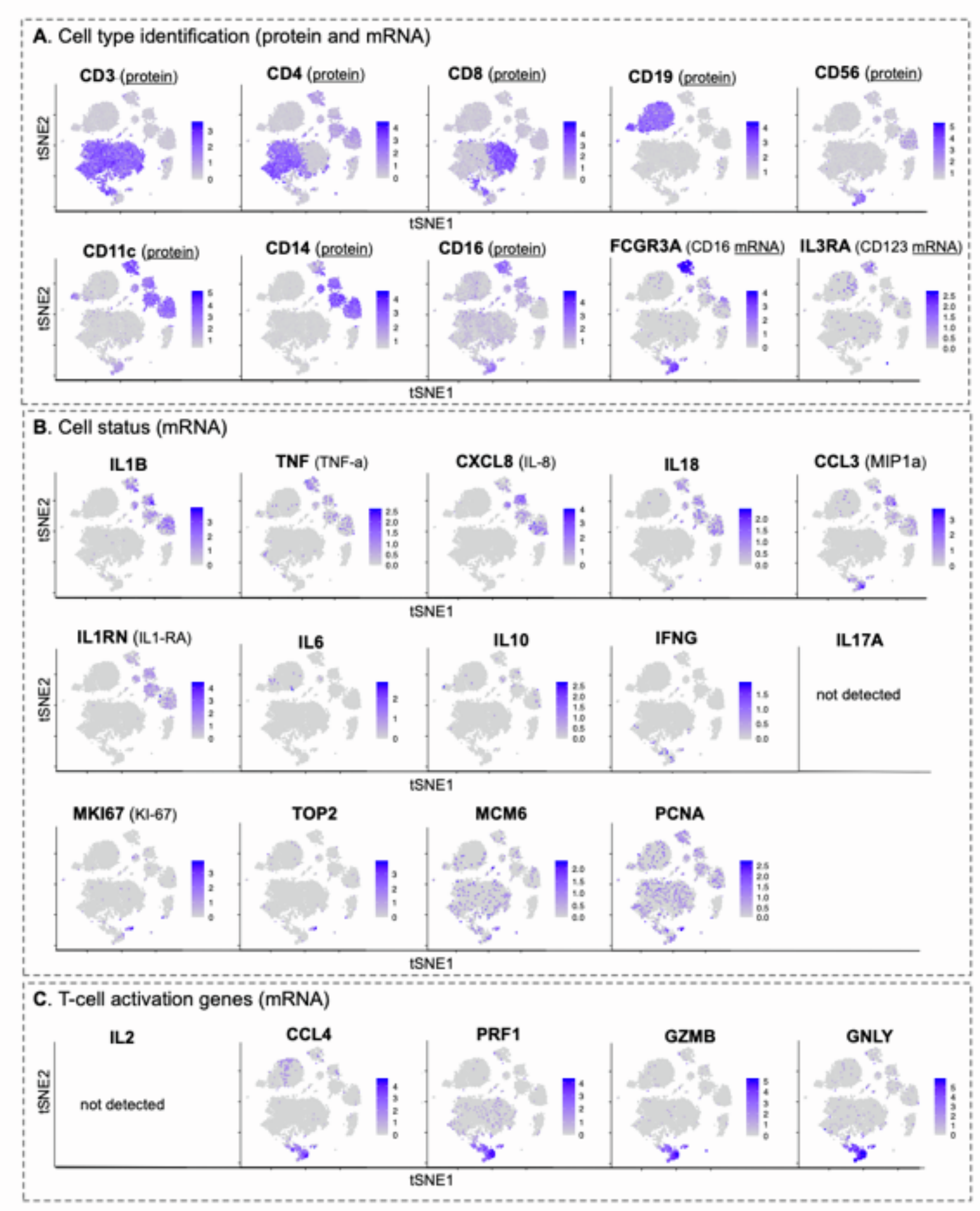

Figure S5. Gene set enrichment analysis of gene expression changes in immune cells from acute MIS-C or KD patients. Related to Figure 2. Heatmap shows the statistical significance of gene set enrichment scores that are comprised of genes that are upregulated (top panel) or downregulated (bottom panel) for each of the indicated immune cell subsets in acute samples from patients KD2, P13, P14 relative to the convalescent sample from P13. The significance of each pathway is indicated by colour.

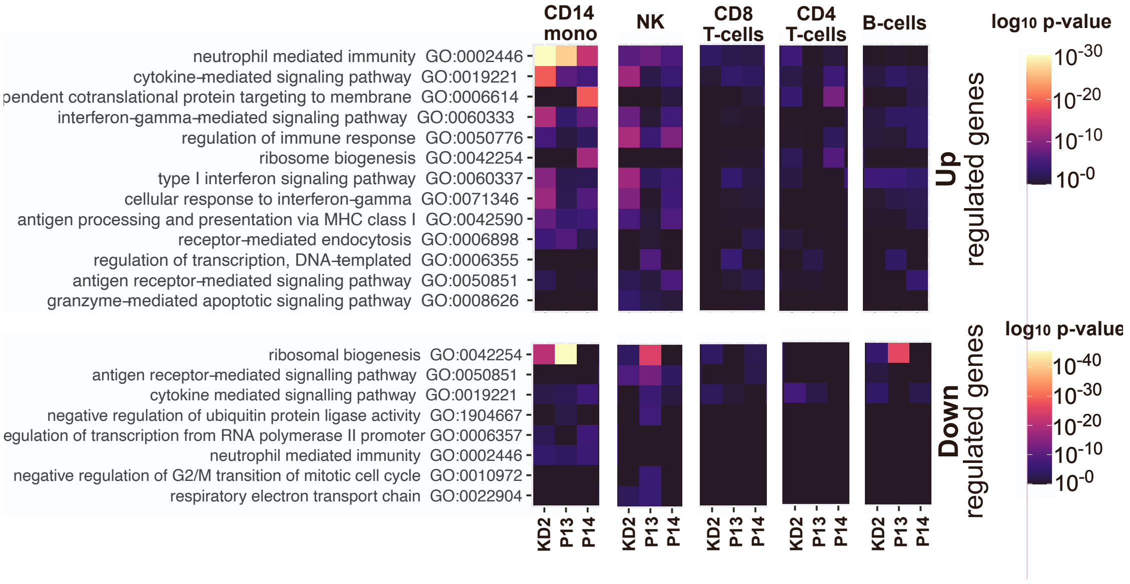

Figure S6. Heatmap showing scaled expression of discriminative genes sets for each of the new clusters generated by reclustering of cells from the originally identified monocyte clusters. Related to Figure 2. Only the top five discriminative genes per cluster are shown.

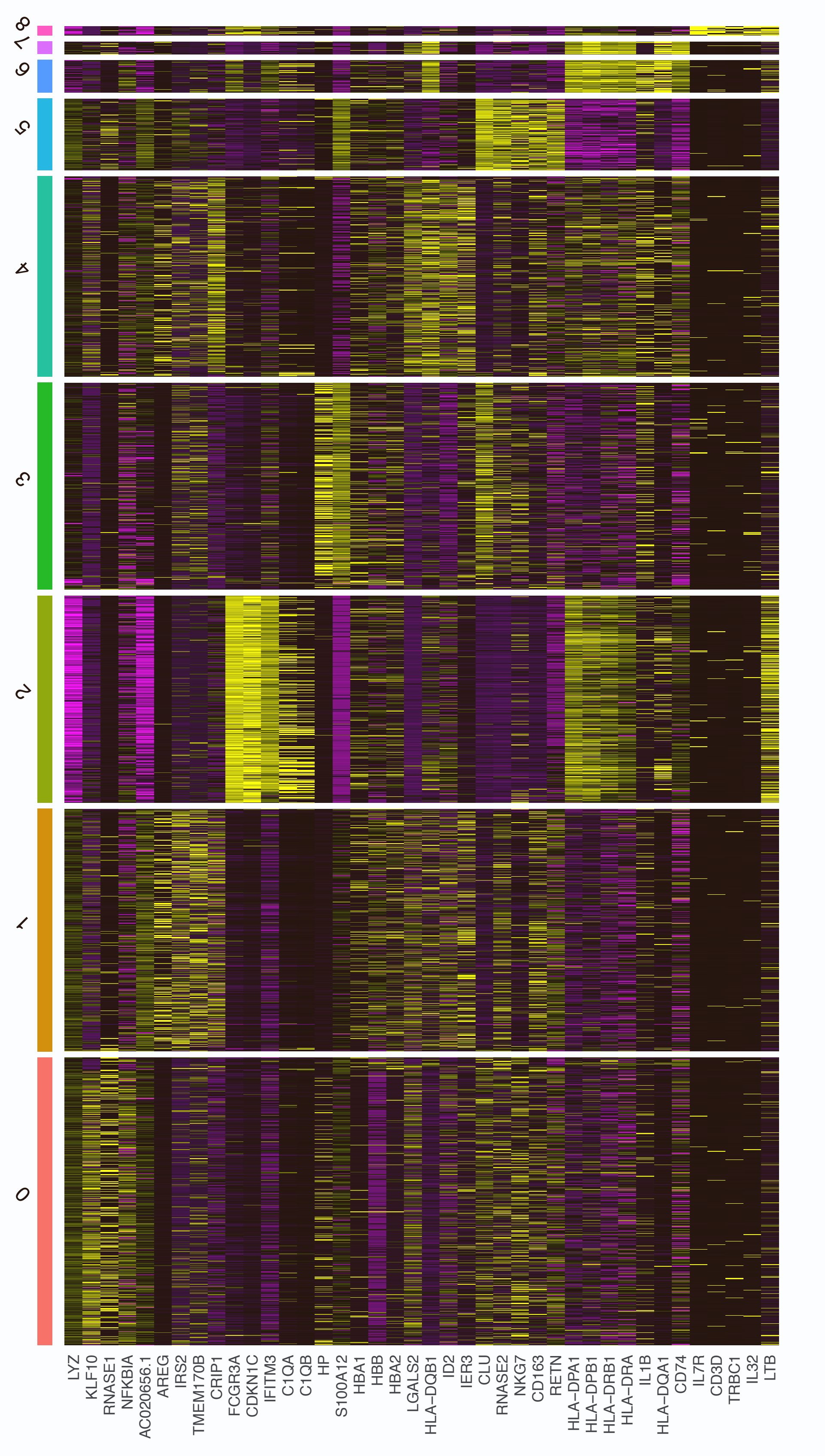

Figure S7. RNA expression and protein levels in patient monocytes. Related to Figure 2C. UMAP representation of monocytes from all four patient samples coloured by the level of RNA per cell or level of protein per cell measured using antibody derived tags.

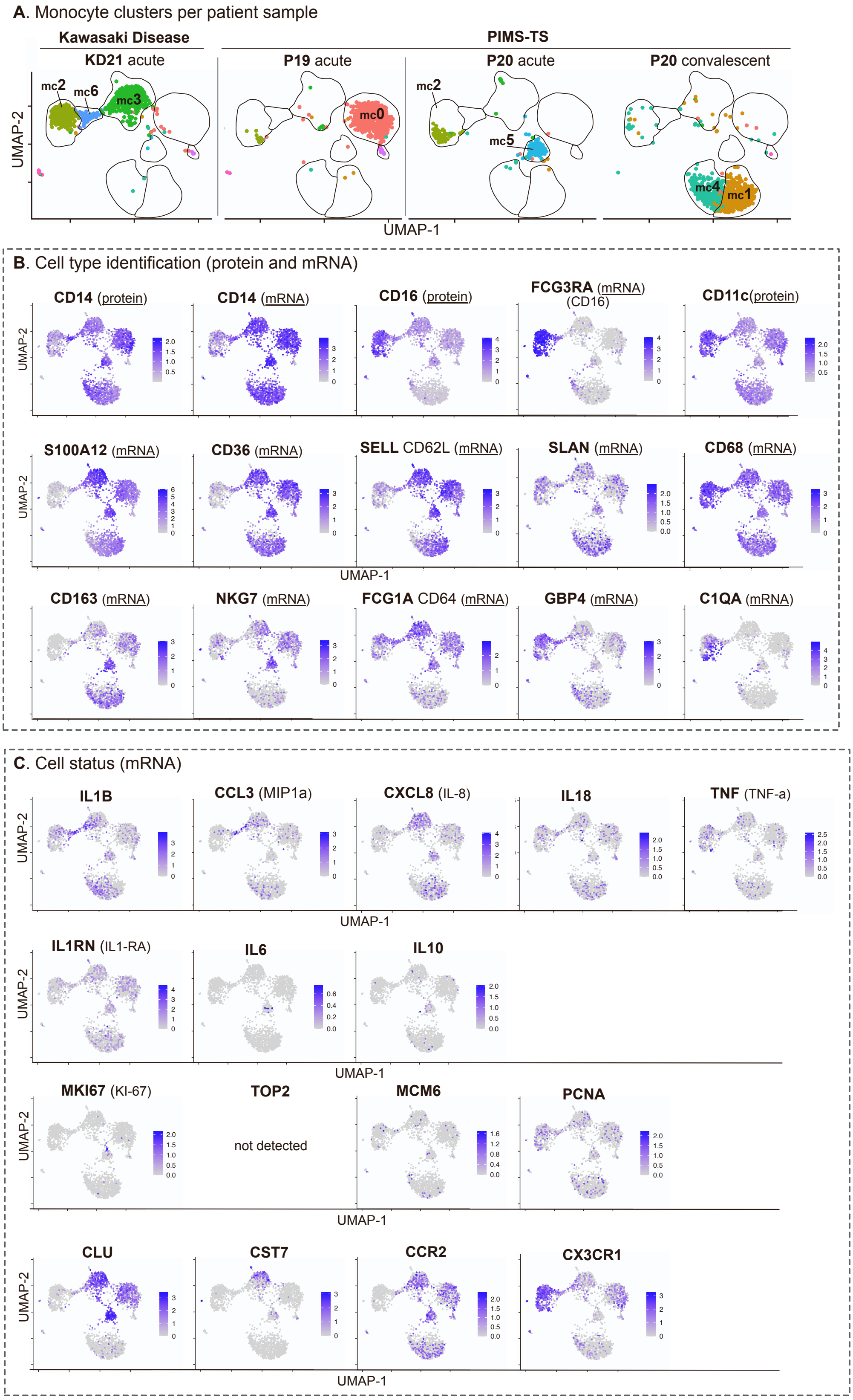

Figure S8. Key events for each patient. Related to Figures 3-7. Length of stay in hospital, treatments received and sample collection timings are shown.

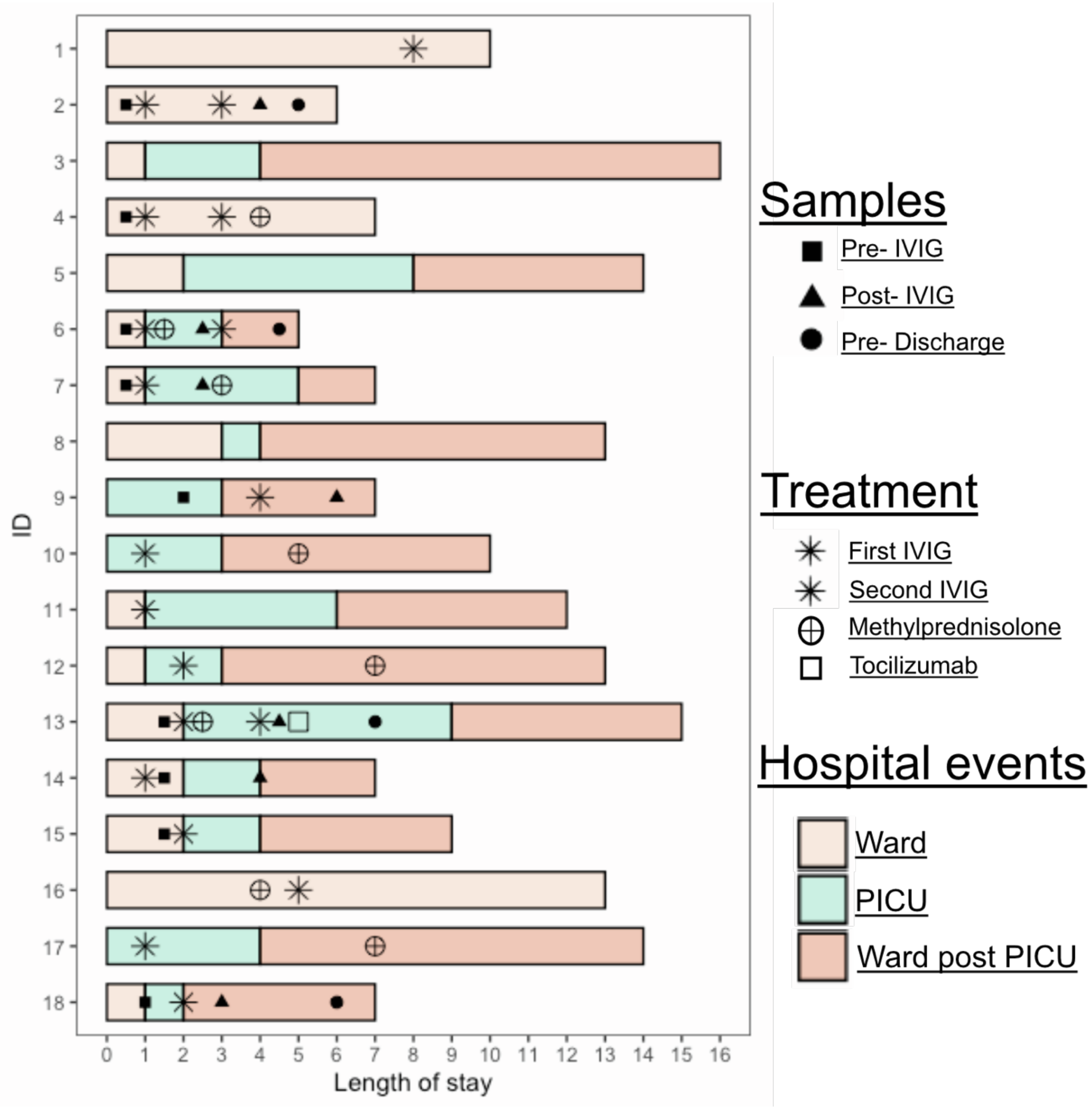

Figure S9. Heatmap showing the median mass intensity of each marker in each of the mononuclear cell clusters. Related to Figures 3,4 and 5. The data shown in the figure was produced from six acute stage MIS-C patients (data from each patient combined together to produce the figure).

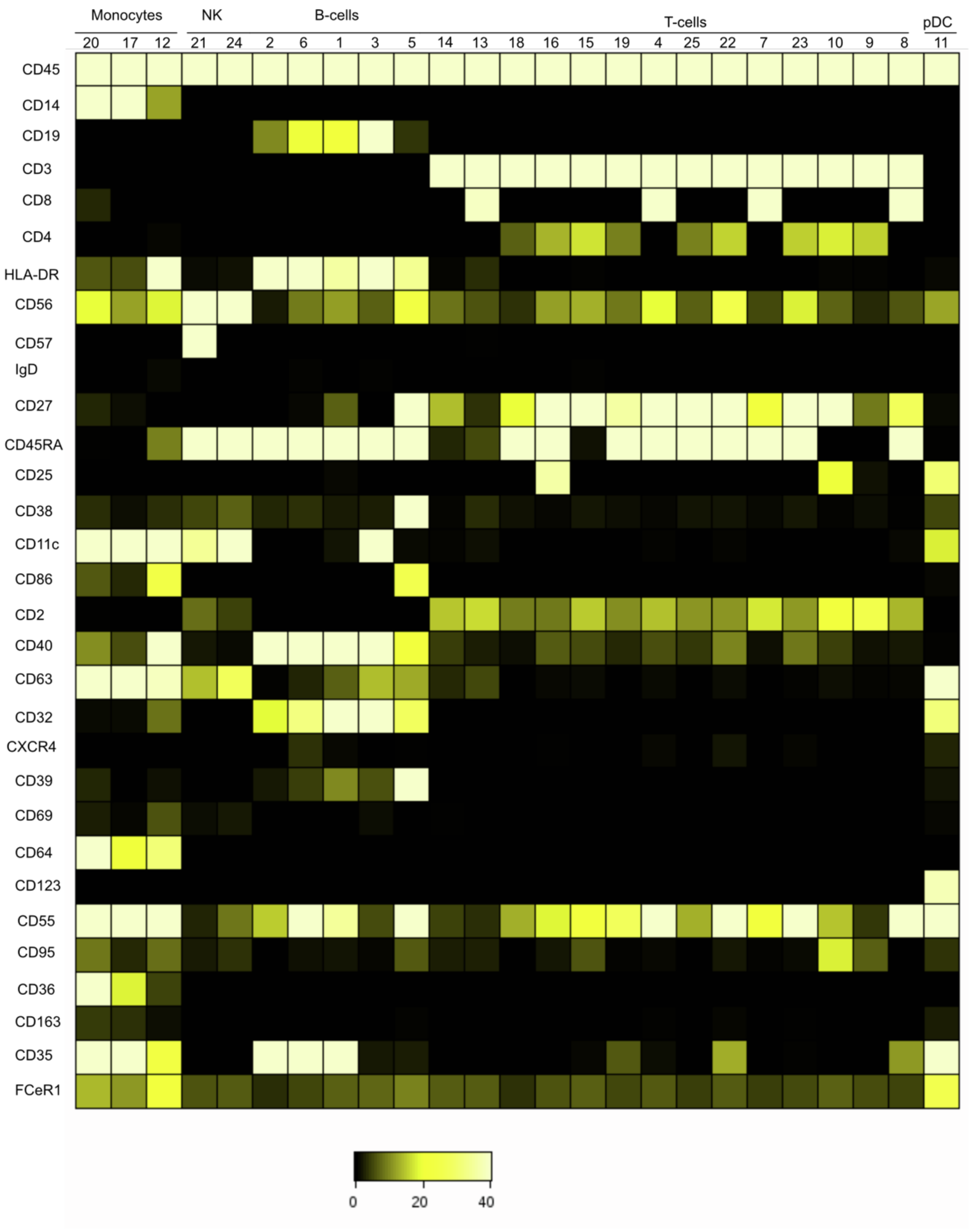

Figure S10. Biaxial plots of CD163 and CD64 expression by metacluster 17 monocytes for each individual. Related to Figure 3. (n=7 healthy donors, 6 MIS-C patients). D/C indicates sample taken at discharge (SI Figure 7).

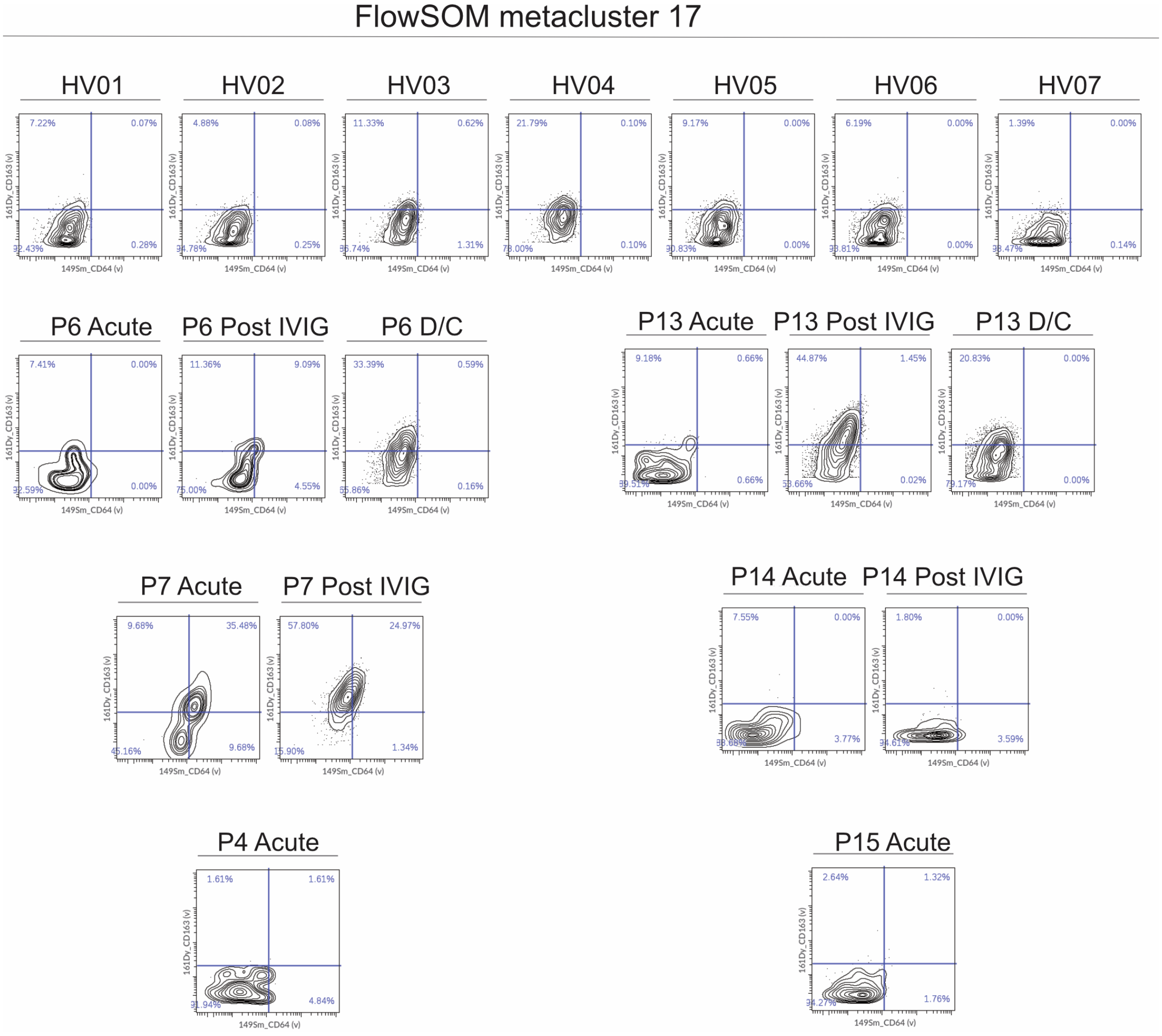

Figure S11. Gating strategy to characterise T-cell differentiation and activation state. Related to Figure 4. Panel A. Gating strategy for major B and T cells subsets. B. Gating strategy for HLA-DR positive cells in the 4 subsets of CD8+ and CD4+ T-cells. C. Frequency of CD4+ and CD8+ T-cells within each of the four subsets for each individual healthy control and MIS-C acute patient. Statistical significance was determined by Wilcoxon test between healthy children and MIS-C patients at the acute stage of disease. ns: no significant difference.

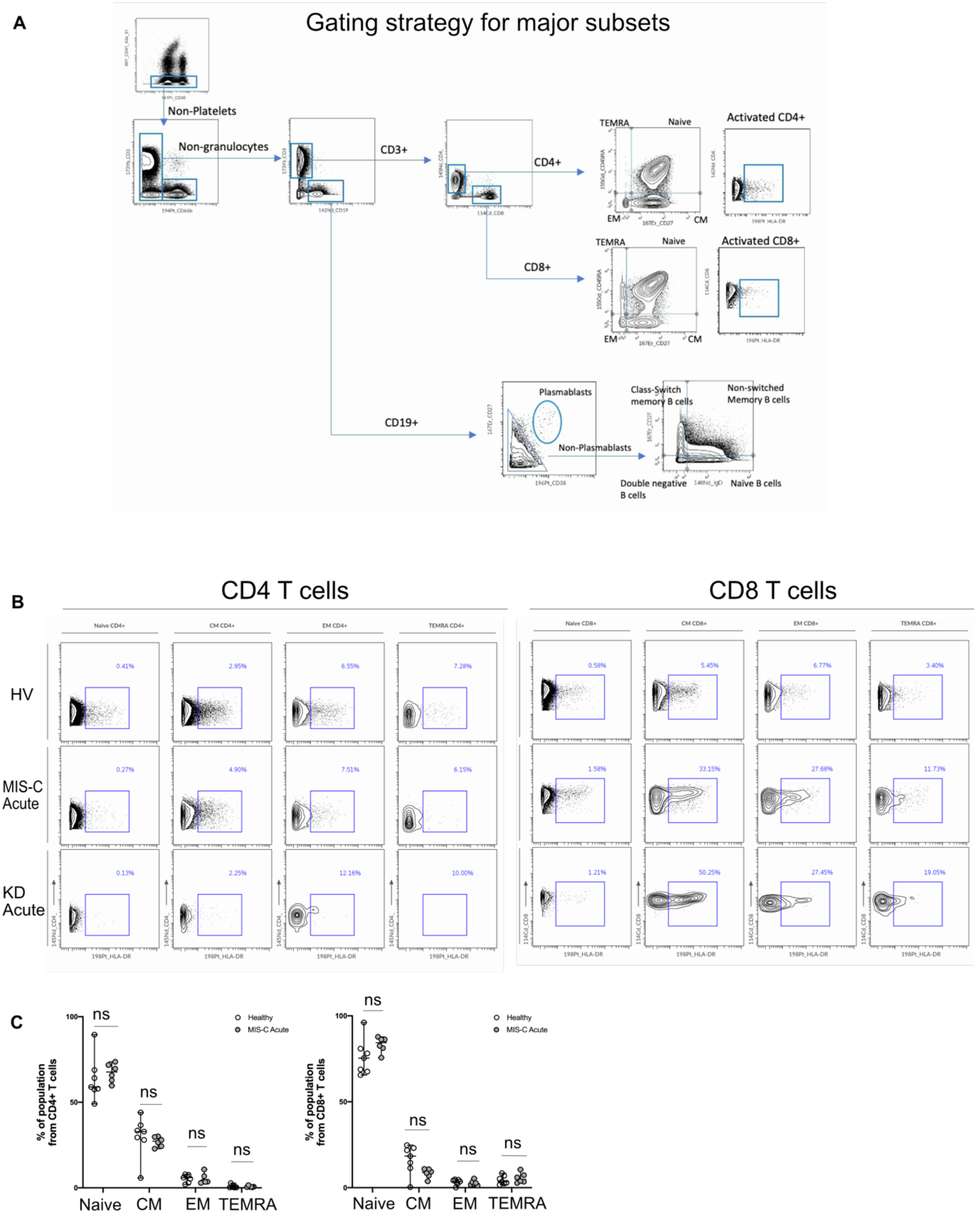

Supplement: Document S1. Figures S1–S11 and Tables S1 and S2 [file mmc1.pdf]
